# Supplementary material for: Colorectal cancer mutational profiles correlate with defined microbial communities in the tumor microenvironment
Source: PLoS Genet. 2018 Jun 20;14(6):e1007376. doi: 10.1371/journal.pgen.1007376 (PMC6028121; doi:10.1371/journal.pgen.1007376)
Supplement: S1 Fig — (PDF) [file pgen.1007376.s017.pdf]

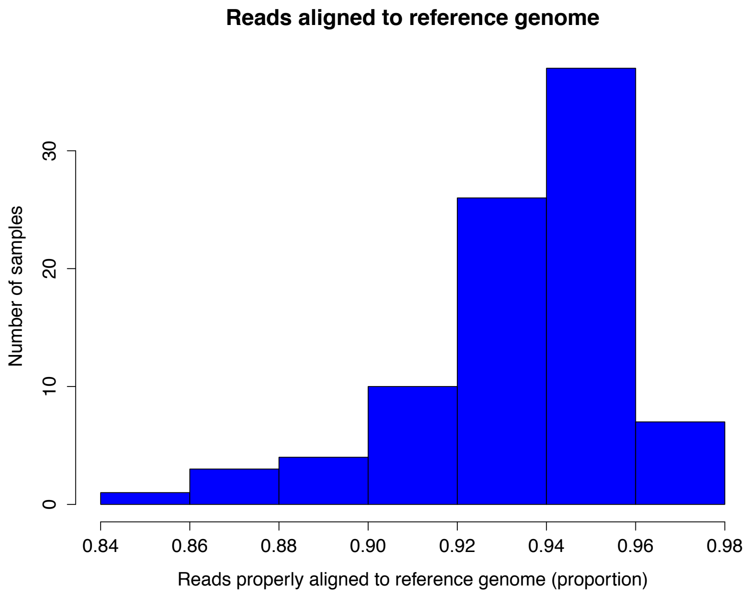


S1 Fig. Histogram of the proportion of reads passing quality filter that aligned to the references genome.
