# Supplementary material for: Colorectal cancer mutational profiles correlate with defined microbial communities in the tumor microenvironment
Source: PLoS Genet. 2018 Jun 20;14(6):e1007376. doi: 10.1371/journal.pgen.1007376 (PMC6028121; doi:10.1371/journal.pgen.1007376)
Supplement: S2 Fig — The histograms at top indicate the frequency distribution of the total (green), missense (blue), and LoF (red) mutations found in the tumors from our patient cohort. The plots at bottom show the absolute numbers of tumor-specific mutations detected in the tumors on a flat scale (bottom left) as well as on a log scale (bottom right). (PDF) [file pgen.1007376.s018.pdf]

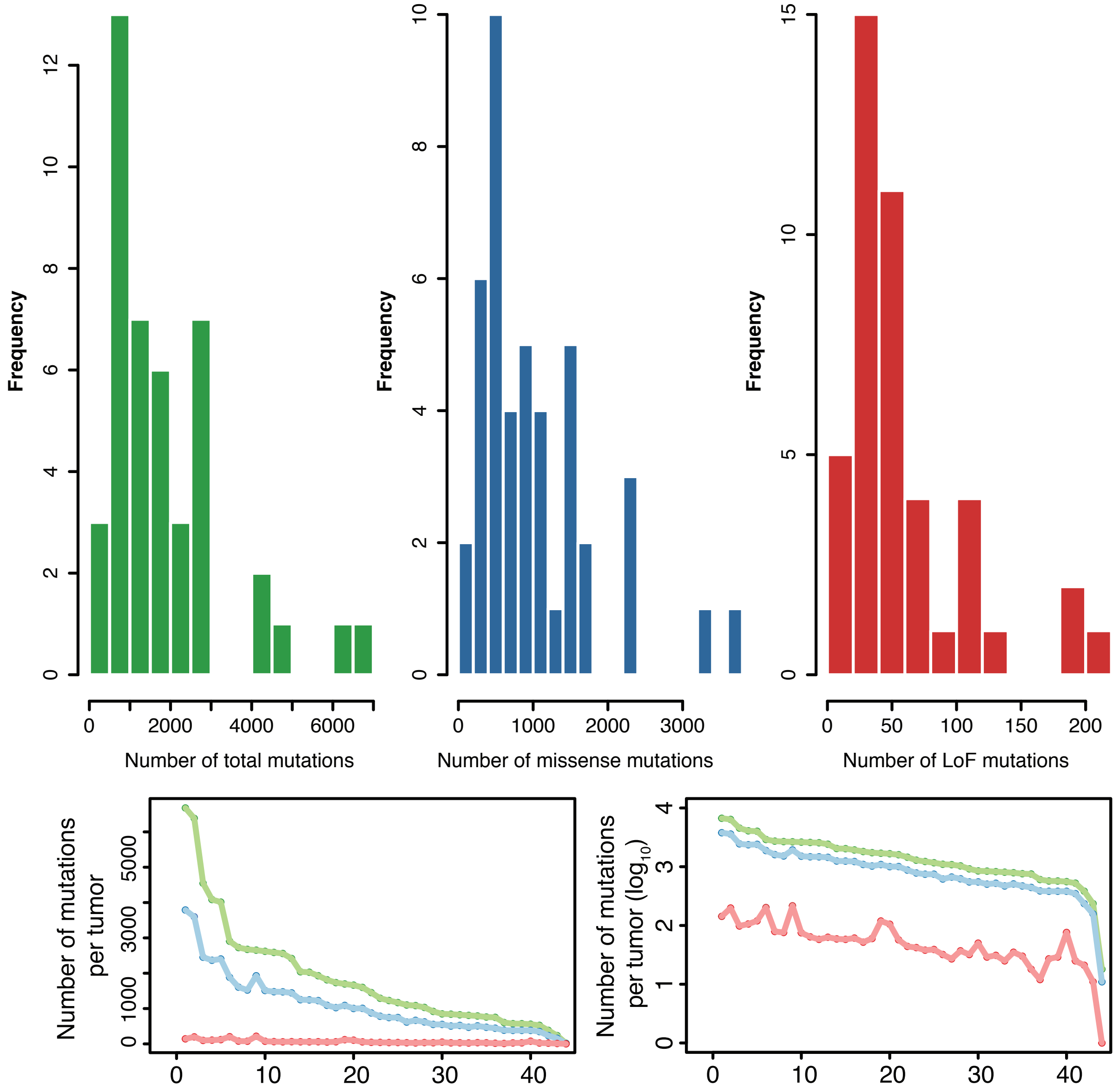

S2 Fig. Tumors harbored a wide range of mutations. The histograms at top indicate the frequency distribution of the total (green), missense (blue), and LoF (red) mutations found in the tumors from our patient cohort. The plots at bottom show the absolute numbers of tumor-specific mutations detected in the tumors on a flat scale (bottom left) as well as on a log scale (bottom right).
