# Supplementary material for: Colorectal cancer mutational profiles correlate with defined microbial communities in the tumor microenvironment
Source: PLoS Genet. 2018 Jun 20;14(6):e1007376. doi: 10.1371/journal.pgen.1007376 (PMC6028121; doi:10.1371/journal.pgen.1007376)
Supplement: S3 Fig — (PDF) [file pgen.1007376.s019.pdf]

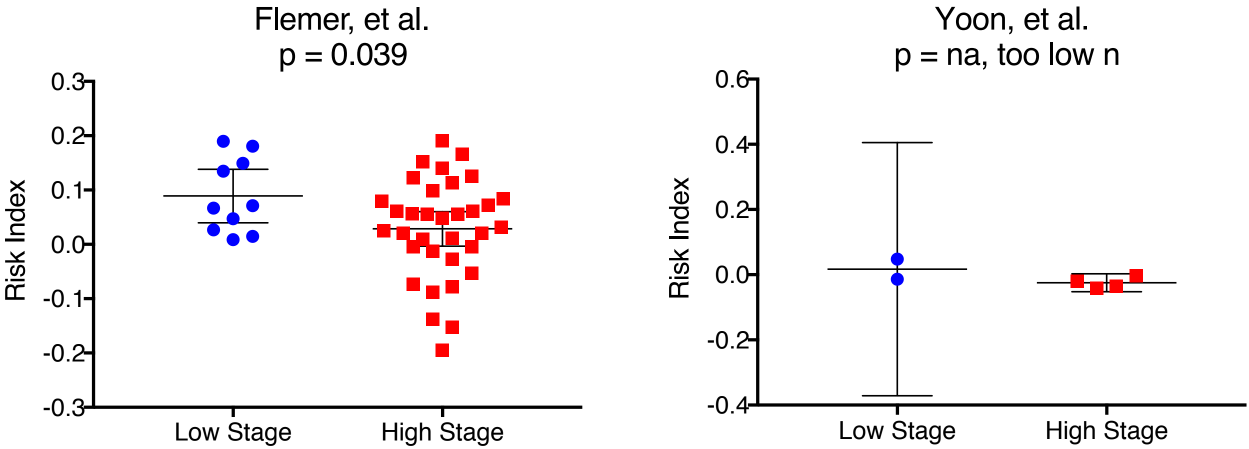

S3 Fig. Column dot plots of the risk indices determined using the stage-relevant model generated in this work to the microbiome data from Flemer, et al. (left) and Yoon, et al. (right).
