# Supplementary material for: Colorectal cancer mutational profiles correlate with defined microbial communities in the tumor microenvironment
Source: PLoS Genet. 2018 Jun 20;14(6):e1007376. doi: 10.1371/journal.pgen.1007376 (PMC6028121; doi:10.1371/journal.pgen.1007376)
Supplement: S4 Fig — Normals included in this model and figure are from healthy individuals from an independent study, not from patient matched samples. (PDF) [file pgen.1007376.s020.pdf]

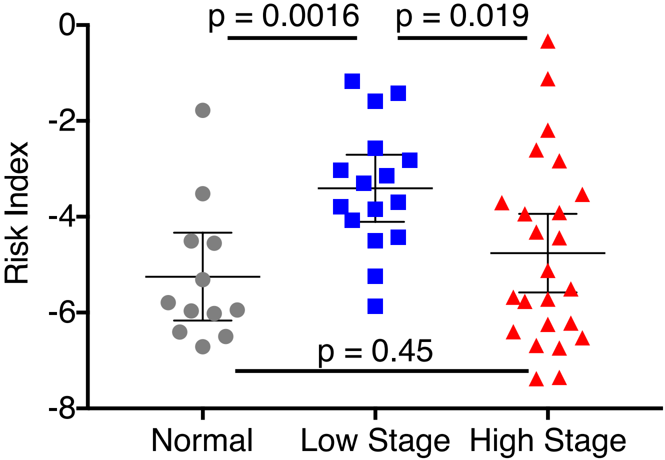

S4 Fig. Column dot plots of the risk indices calculated using a new model designed to separate microbial communities from normal, low stage, and high stage tumors. Normals included in this model and figure are from healthy individuals from an independent study, not from patient matched samples.
