# Supplementary material for: Colorectal cancer mutational profiles correlate with defined microbial communities in the tumor microenvironment
Source: PLoS Genet. 2018 Jun 20;14(6):e1007376. doi: 10.1371/journal.pgen.1007376 (PMC6028121; doi:10.1371/journal.pgen.1007376)
Supplement: S6 Fig — (A) Scaled taxon abundances (columns) in the tumor samples that harbor LoF mutations in the KEGG pathways indicated (rows). Clusters 1 and 2 are labeled to facilitate discussion in main text. (B) Scaled differences (tumor abundance—matched normal abundance) patients that harbor tumor-specific LoF mutations in the KEGG pathways indicated (rows). (PDF) [file pgen.1007376.s022.pdf]

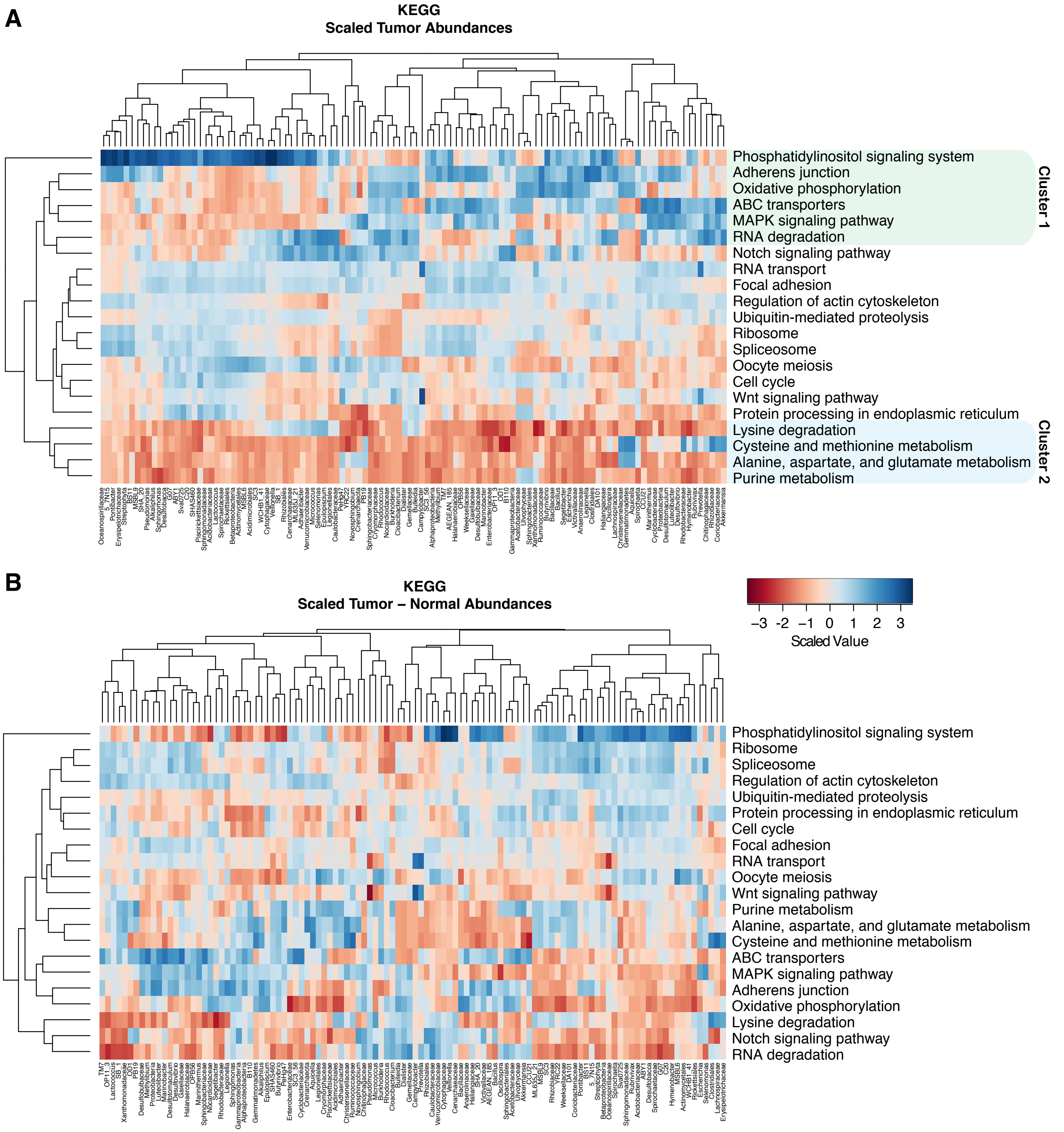

S6 Fig. Heatmaps demonstrating the patterns of microbial abundances for patient samples with prevalent LoF mutations in KEGG pathways. (A) Scaled taxon abundances (columns) in the tumor samples that harbor LoF mutations in the KEGG pathways indicated (rows). Clusters 1 and 2 are labeled to facilitate discussion in main text. (B) Scaled differences (tumor abundance - matched normal abundance) patients that harbor tumor-specific LoF mutations in the KEGG pathways indicated (rows).
