# Supplementary material for: Colorectal cancer mutational profiles correlate with defined microbial communities in the tumor microenvironment
Source: PLoS Genet. 2018 Jun 20;14(6):e1007376. doi: 10.1371/journal.pgen.1007376 (PMC6028121; doi:10.1371/journal.pgen.1007376)
Supplement: S8 Fig — (PDF) [file pgen.1007376.s024.pdf]

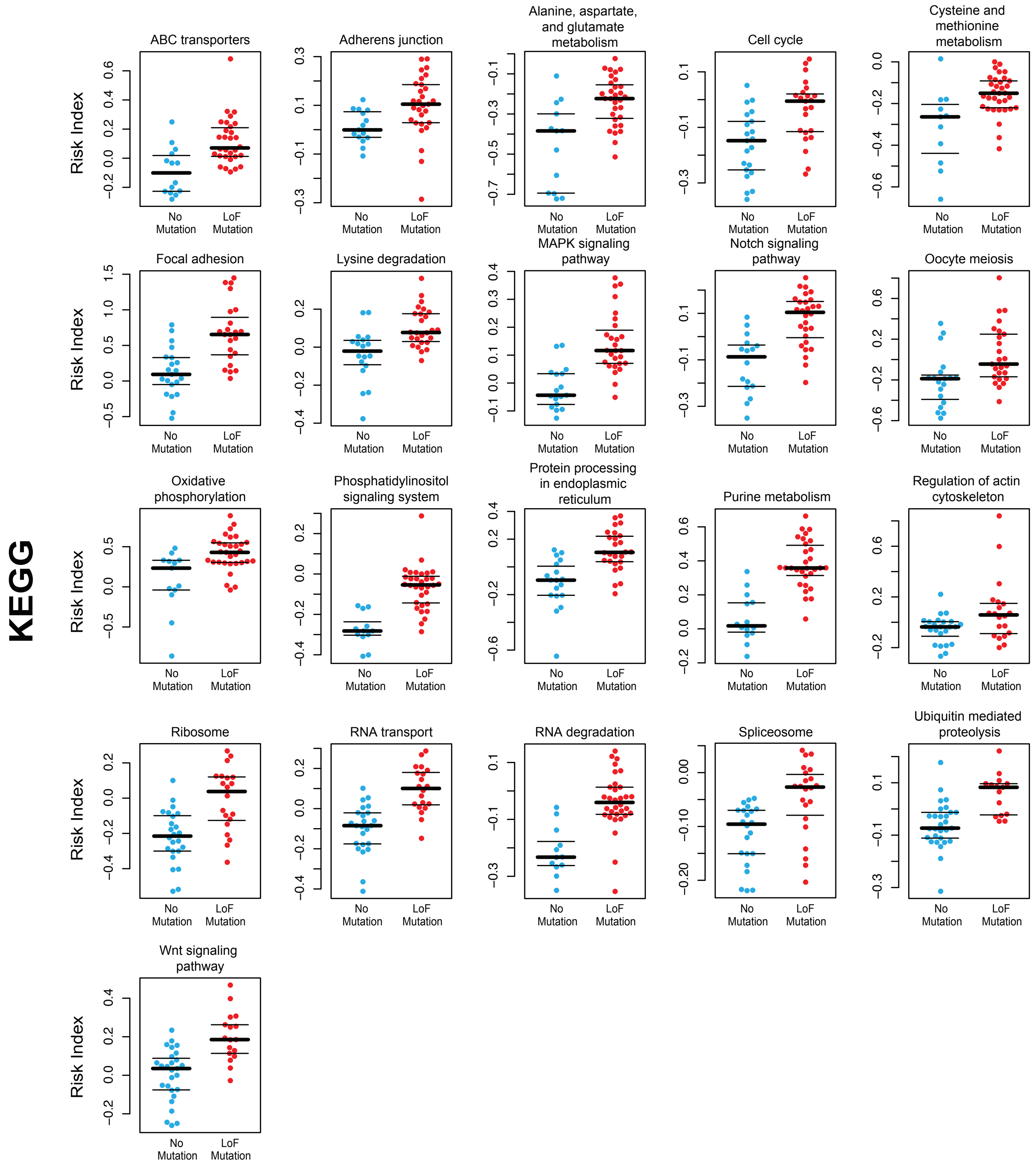


S8 Fig. LoF mutations in KEGG pathways can be predicted using a risk index as a classifier (y-axis).
