# Supplementary figures and images for: Colorectal cancer mutational profiles correlate with defined microbial communities in the tumor microenvironment
Source: PLoS Genet. 2018 Jun 20;14(6):e1007376. doi: 10.1371/journal.pgen.1007376 (PMC6028121; doi:10.1371/journal.pgen.1007376)

# PID

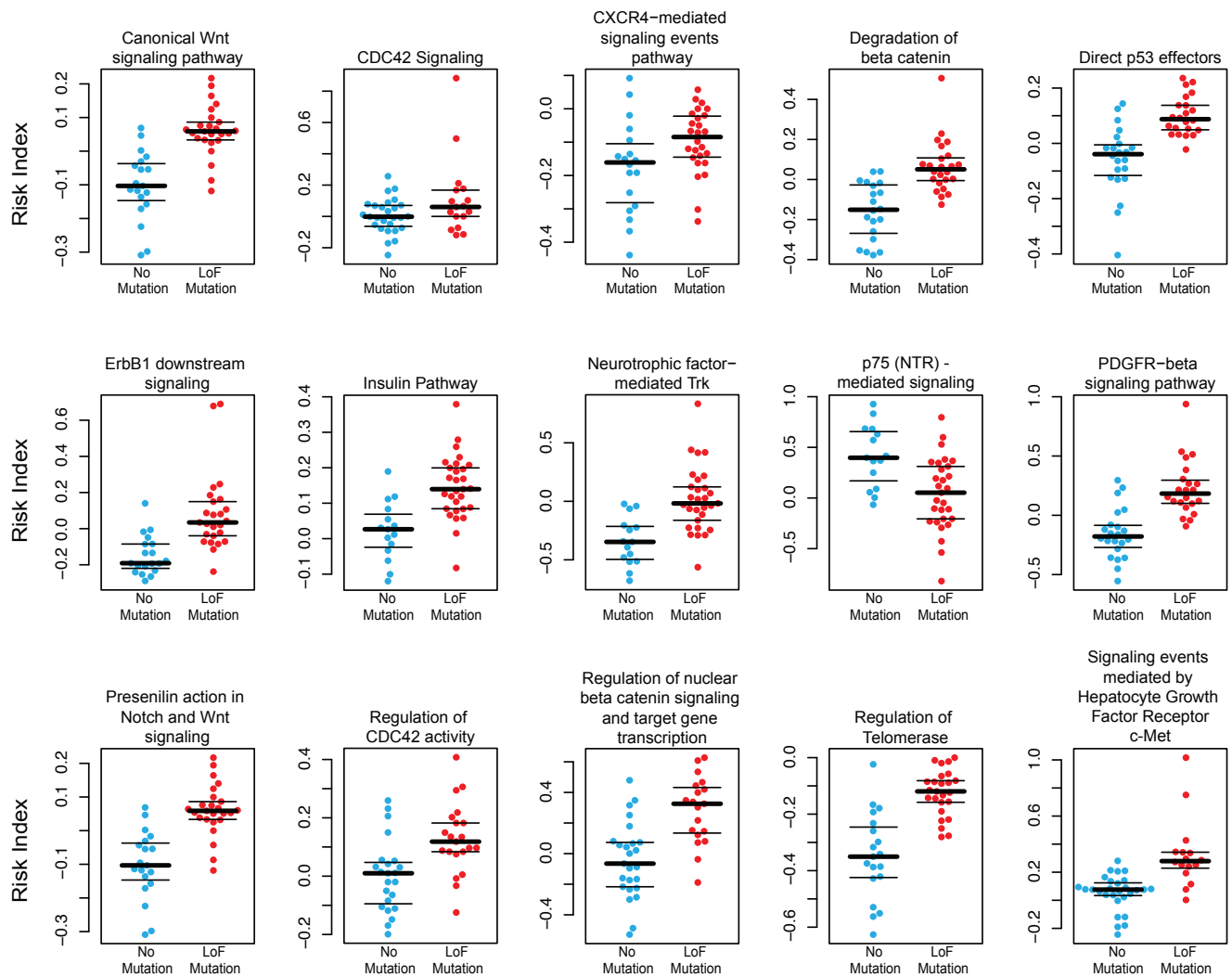

S10 Fig. LoF mutations in PID pathways can be predicted using a risk index as a classifier (y-axis).

Supplement: S10 Fig — (PDF) [file pgen.1007376.s026.pdf]
