# Supplementary material for: Colorectal cancer mutational profiles correlate with defined microbial communities in the tumor microenvironment
Source: PLoS Genet. 2018 Jun 20;14(6):e1007376. doi: 10.1371/journal.pgen.1007376 (PMC6028121; doi:10.1371/journal.pgen.1007376)
Supplement: S11 Fig — The average of the 10-fold cross-validation curves is represented as a thick black line. The AUC are indicated at bottom of each pathway panel. This figure also includes BARD1 and Class I PI3K pathways, for references, although neither of these achieved statistical significance in other tests. (PDF) [file pgen.1007376.s027.pdf]

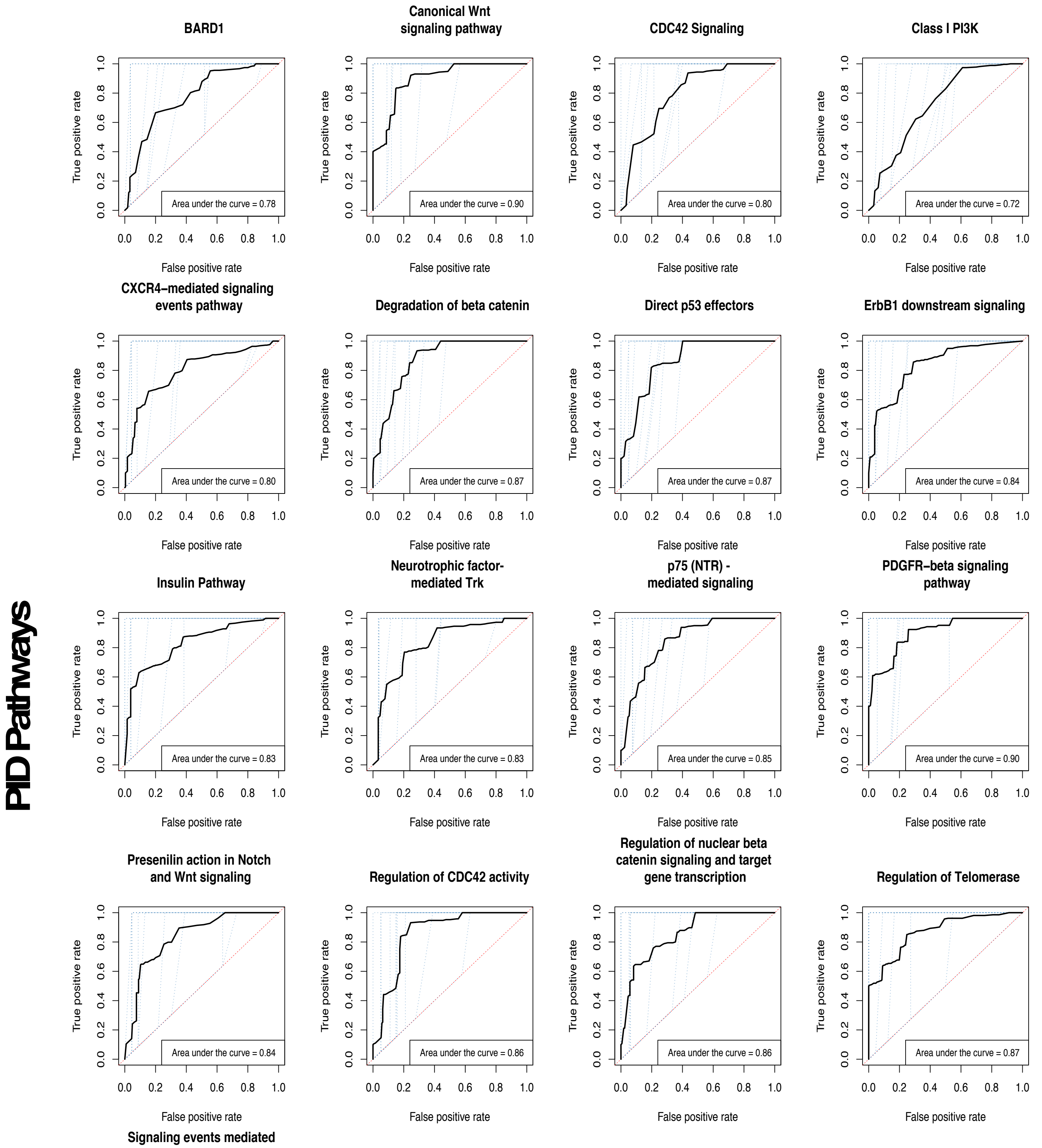

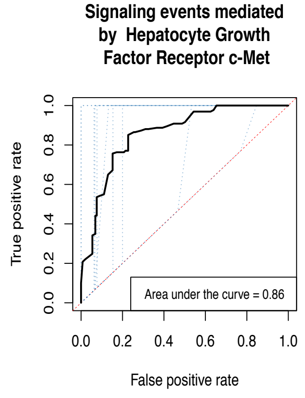

S11 Fig. ROC curves were generated for each of the PID pathways indicated in S10 Fig using a 10-fold cross- validation (blue dotted lines). The average of the 10-fold cross-validation curves is represented as a thick black line. The AUC are indicated at bottom of each pathway panel. This figure also includes BARD1 and Class I PI3K pathways, for references, although neither of these achieved statistical significance in other tests.
