# Supplementary material for: Colorectal cancer mutational profiles correlate with defined microbial communities in the tumor microenvironment
Source: PLoS Genet. 2018 Jun 20;14(6):e1007376. doi: 10.1371/journal.pgen.1007376 (PMC6028121; doi:10.1371/journal.pgen.1007376)

S12 Fig. Large set of abundance plots for the taxa from each of the genes, KEGG pathways, and PID pathways that harbored prevalent LoF mutations and showed significance as a means of predicting the interaction between the mutation and the microbiota. Abundances are plotted as both column dot plots as well as horizontal bar plots of the differences in the mean abundances of a subset of the taxa predicted to interact differentially with tumors with a LoF mutation relative to those without the indicated mutation. These subsets represent those taxa that had a mean difference in abundance of greater than 0.1%, proportionally.

S12 Fig | Abundance Barcharts.

Genes

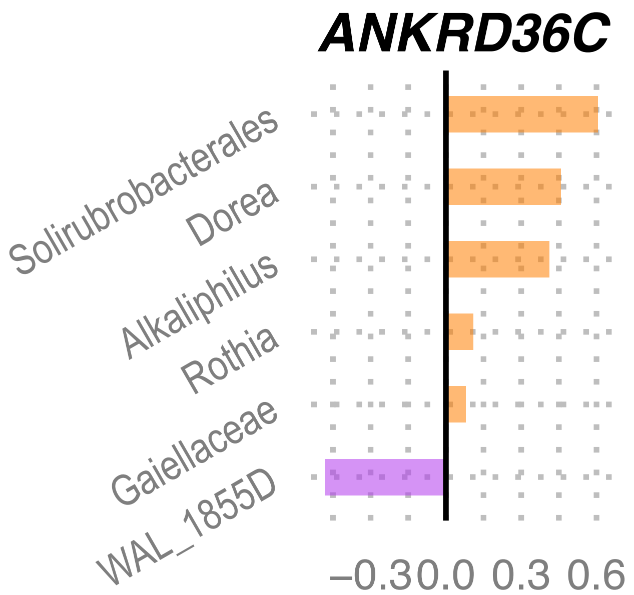

ANKRD36C
Solirubrobacterales
Dorea
Alkaliphilus
Rothia
Gaiellaceae
WAL_1855D
−0.3
0.0
0.3
0.6

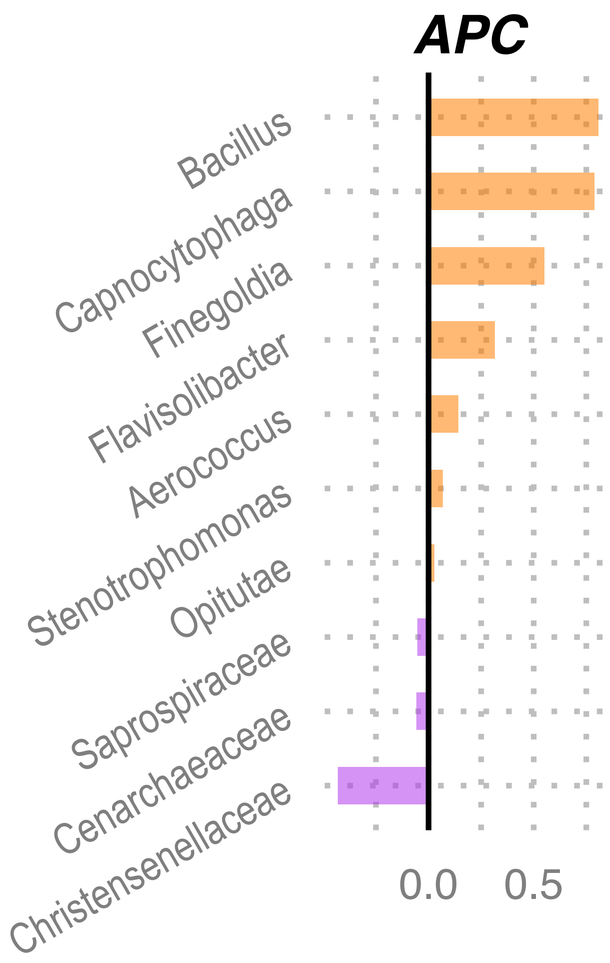

APC
Bacillus
Capnocytophaga
Finegoldia
Flavisolibacter
Aerococcus
Stenotrophomonas
Opitutae
Saprospiraceae
Cenarchaeaceae
Christensenellaceae
0.0
0.5

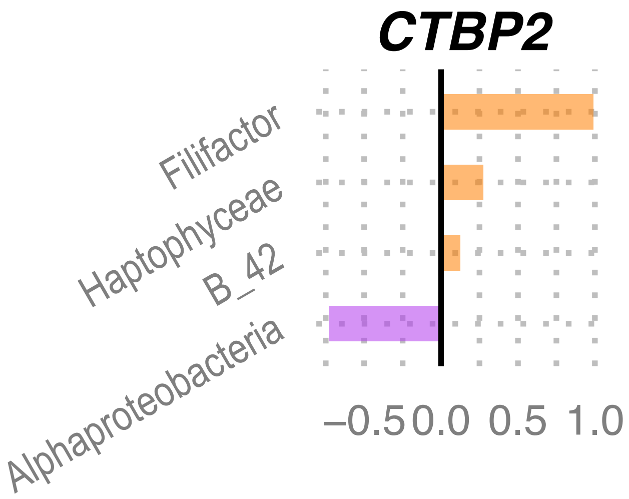
CTBP2
Filifactor
Haptophyceae
B_42
Alphaproteobacteria
−0.5
0.0
0.5
1.0

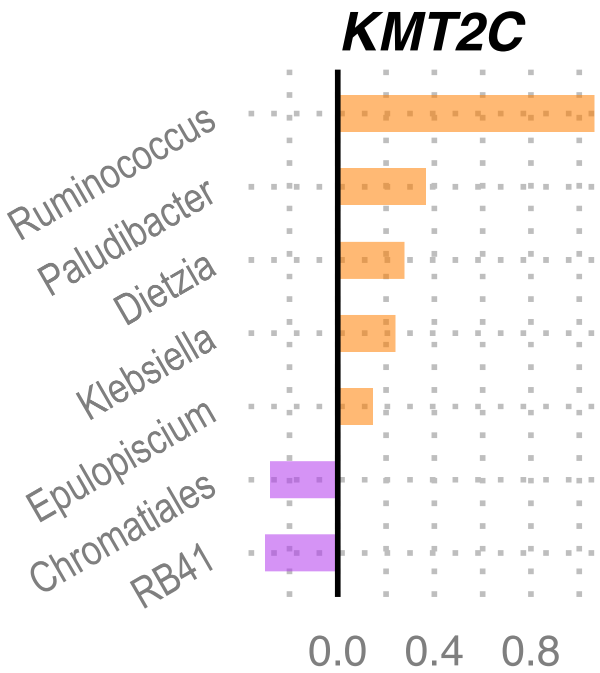
KMT2C
Ruminococcus
Paludibacter
Dietzia
Klebsiella
Epulopiscium
Chromatiales
RB41
0.0
0.4
0.8

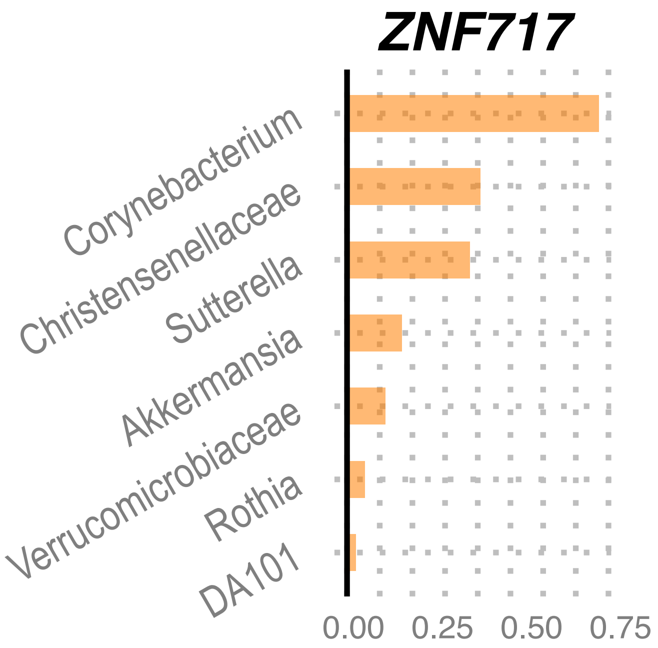
ZNF717
Corynebacterium
Christensenellaceae
Sutterella
Akkermansia
Verrucomicrobiaceae
Rothia
DA101
0.00
0.25
0.50
0.75

S12 Fig I Abundance Barcharts.

KEGG

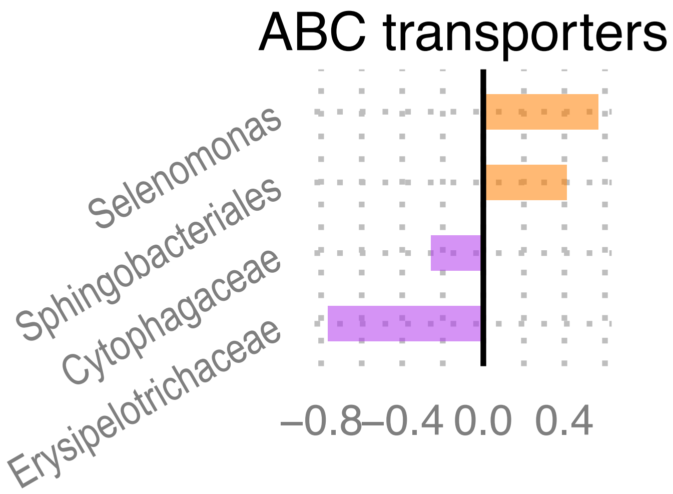

ABC transporters
Selenomonas
Sphingobacteriales
Cytophagaceae
Erysipelotrichaceae
−0.8
−0.4
0.0
0.4

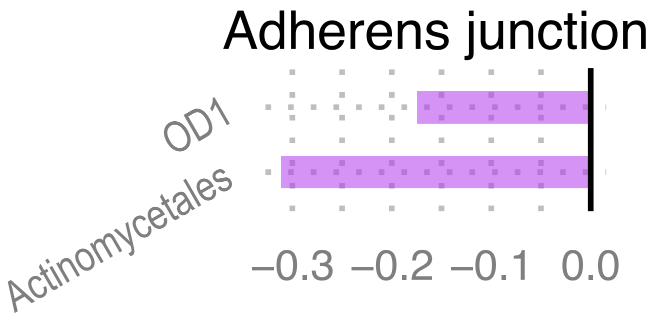

Adherens junction
OD1
Actinomycetales
−0.3
−0.2
−0.1
0.0

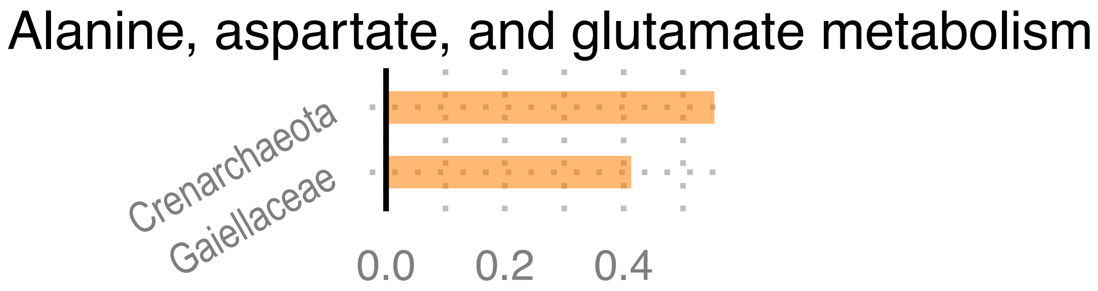

Alanine, aspartate, and glutamate metabolism
Crenarchaeota
Gaiellaceae
0.0
0.2
0.4

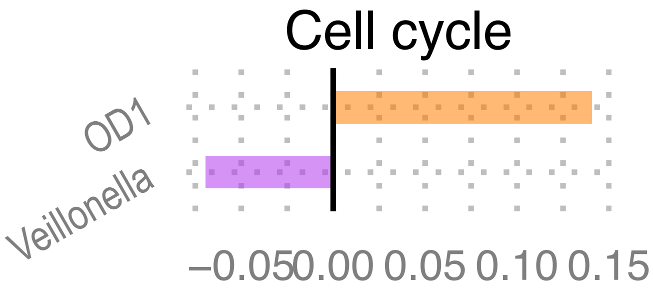

Cell cycle
OD1
Veillonella
−0.05
0.00
0.05
0.10
0.15

Cysteine and methionine metabolism

File intentionally blank -no taxa met the abundance-difference threshold for inclusion

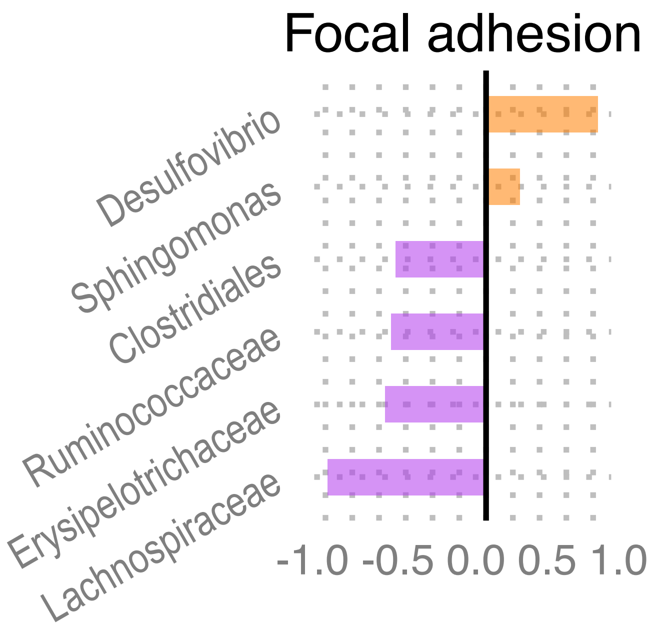
Focal adhesion
Desulfovibrio
Sphingomonas
Clostridiales
Ruminococcaceae
Erysipelotrichaceae
Lachnospiraceae
-1.0
-0.5
0.0
0.5
1.0

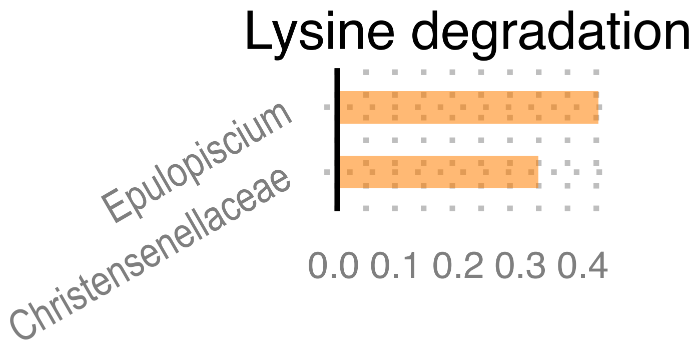
Lysine degradation
Epulopiscium
Christensenellaceae
0.0 0.1 0.2 0.3 0.4

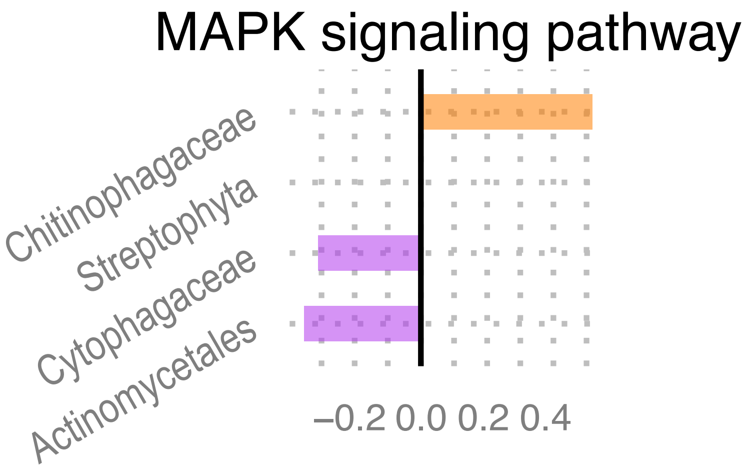
MAPK signaling pathway
Chitinophagaceae
Streptophyta
Cytophagaceae
Actinomycetales
−0.2 0.0 0.2 0.4

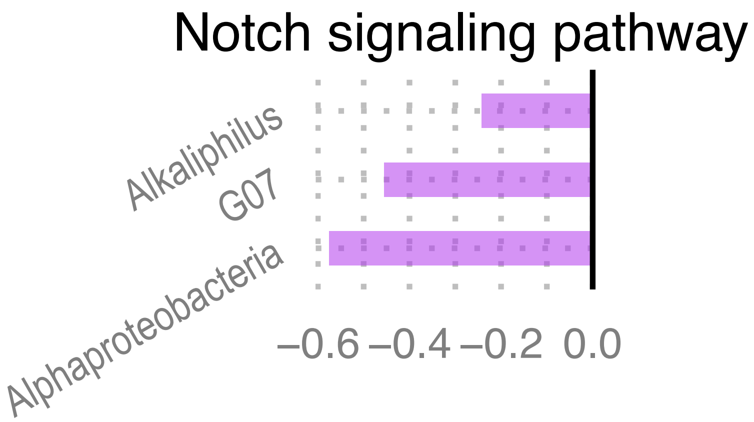

Notch signaling pathway
Alkaliphilus
G07
Alphaproteobacteria
−0.6
−0.4
−0.2
0.0

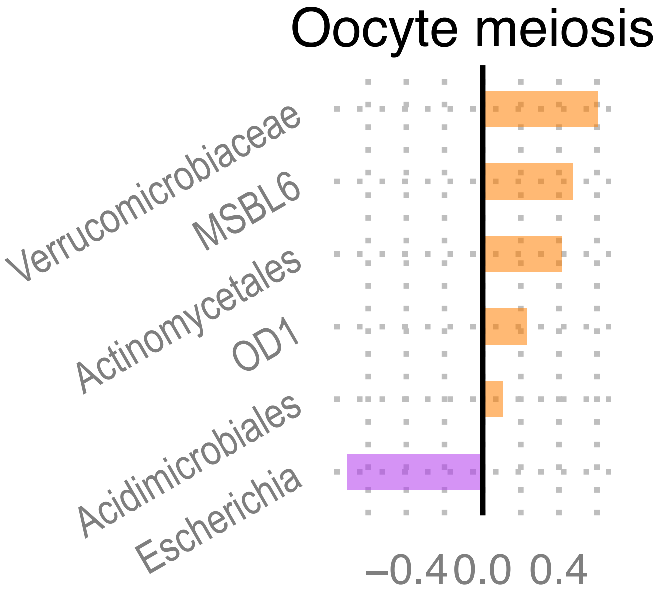

Oocyte meiosis
Verrucomicrobiaceae
MSBL6
Actinomycetales
OD1
Acidimicrobiales
Escherichia
−0.4
0.0
0.4

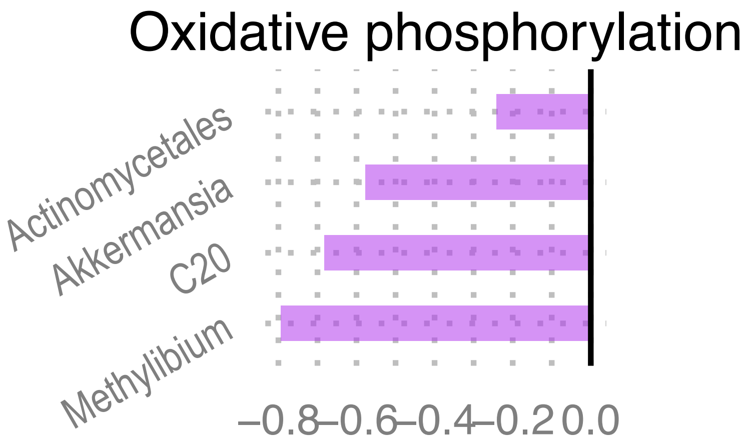

Oxidative phosphorylation
Actinomycetales
Akkermansia
C20
Methylibium
−0.8
−0.6
−0.4
−0.2
0.0

Phosphatidylinositol signaling system

File intentionally blank -no taxa met the abundance-difference threshold for inclusion

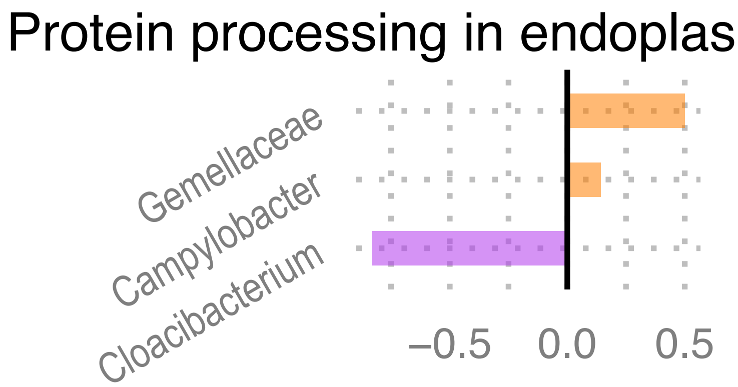

Protein processing in endoplasmic reticulum
Gemellaceae
Campylobacter
Cloacibacterium
−0.5
0.0
0.5

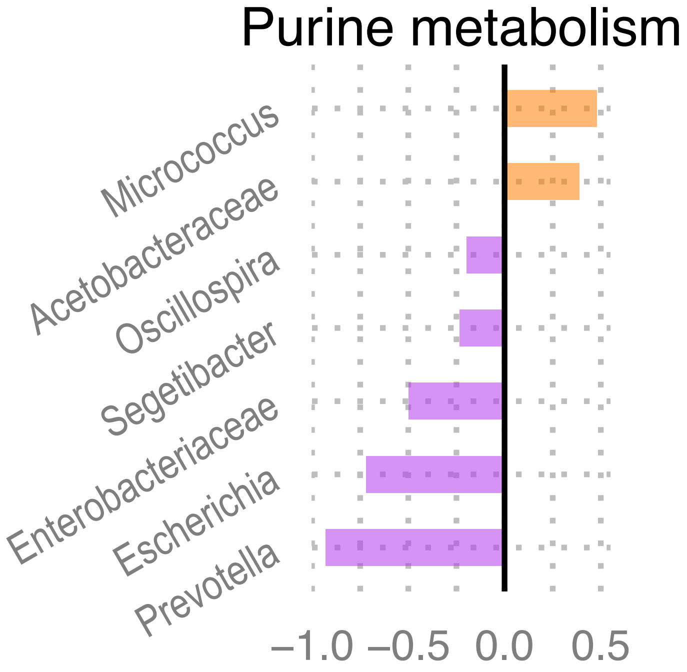

Purine metabolism
Micrococcus
Acetobacteraceae
Oscillospira
Segetibacter
Enterobacteriaceae
Escherichia
Prevotella
−1.0
−0.5
0.0
0.5

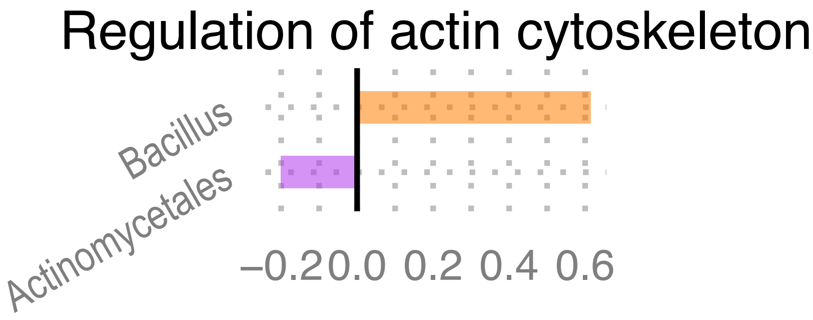

Regulation of actin cytoskeleton
Bacillus
Actinomycetales
−0.2
0.0
0.2
0.4
0.6

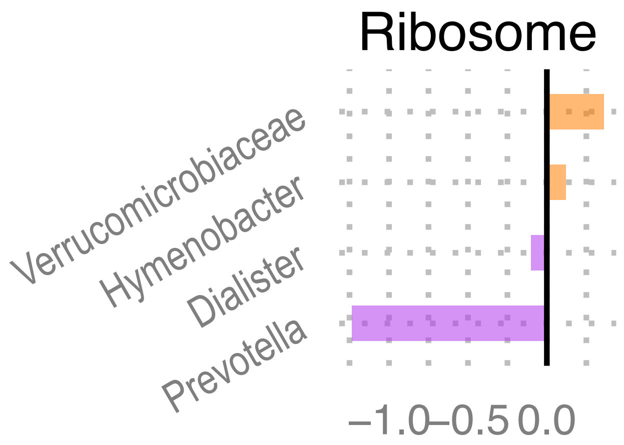

Ribosome
Verrucomicrobiaceae
Hymenobacter
Dialister
Prevotella
−1.0
−0.5
0.0

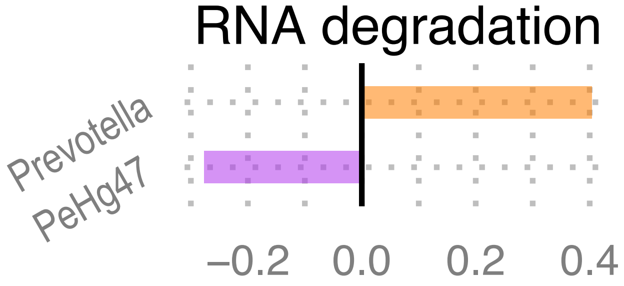
RNA degradation
Prevotella
PeHg47
−0.2
0.0
0.2
0.4

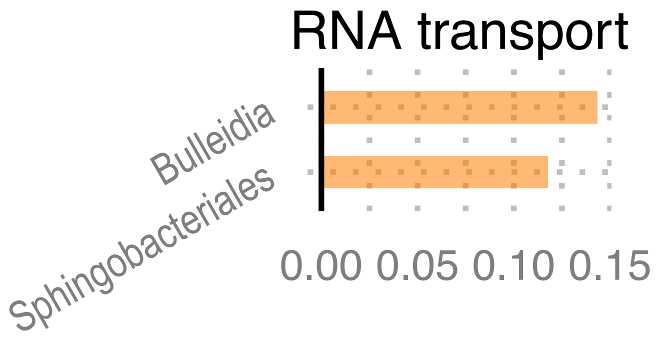

RNA transport
Bulleidia
Sphingobacteriales
0.00
0.05
0.10
0.15

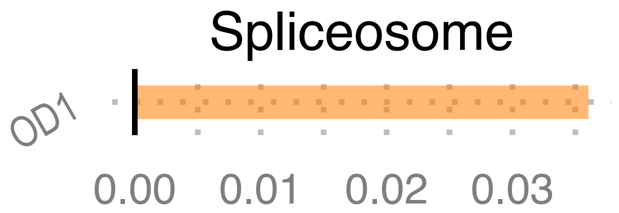
Spliceosome
OD1
0.00
0.01
0.02
0.03

Ubiquitin mediated proteolysis

File intentionally blank -no taxa met the abundance-difference threshold for inclusion

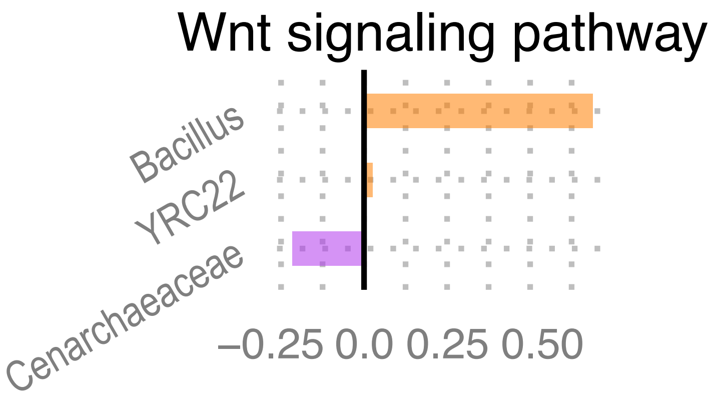

Wnt signaling pathway
Bacillus
YRC22
Cenarchaeaceae
−0.25
0.0
0.25
0.50

S12 Fig I Abundance Barcharts.

PID

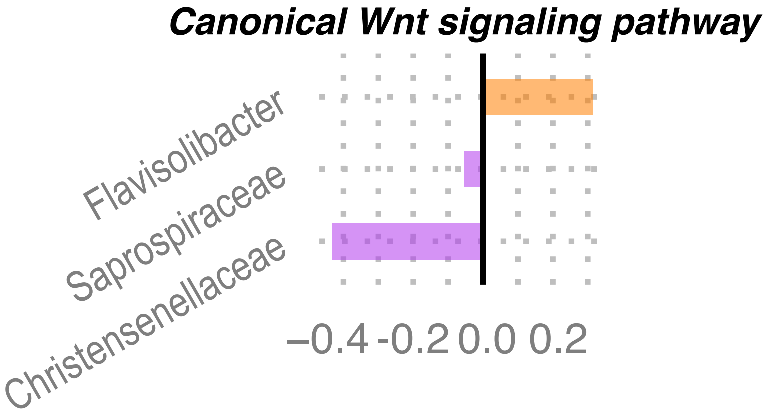

Canonical Wnt signaling pathway
Flavisolibacter
Saprospiraceae
Christensenellaceae
–0.4
-0.2
0.0
0.2

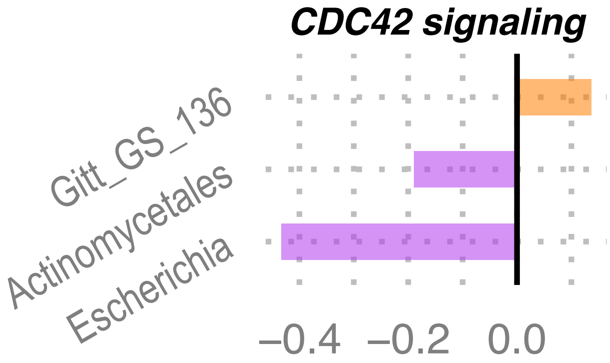

CDC42 signaling
Gitt_GS_136
Actinomycetales
Escherichia
−0.4
−0.2
0.0

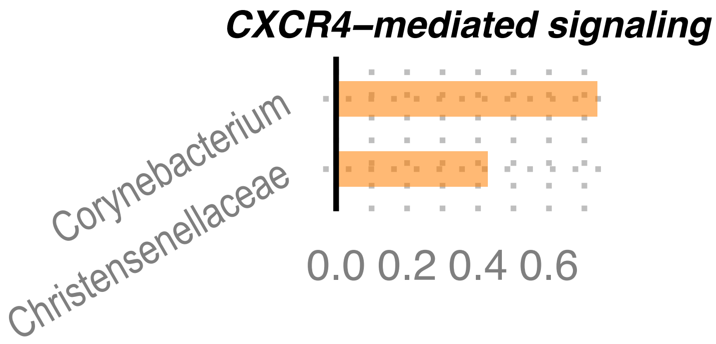

CXCR4–mediated signaling
Corynebacterium
Christensenellaceae
0.0
0.2
0.4
0.6

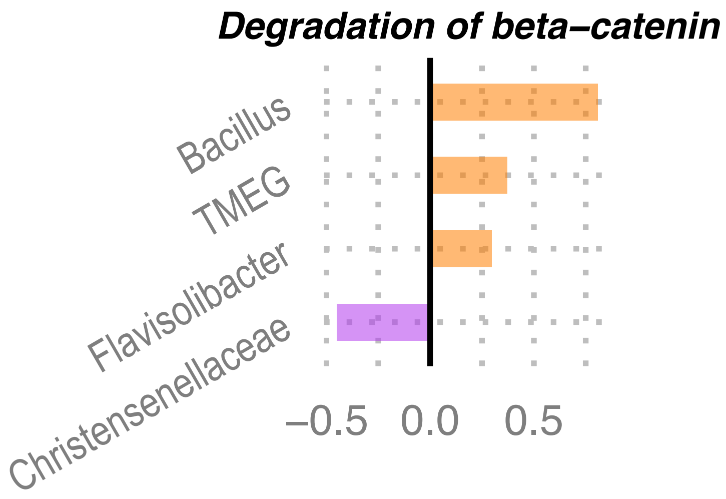

Degradation of beta−catenin
Bacillus
TMEG
Flavisolibacter
Christensenellaceae
−0.5
0.0
0.5

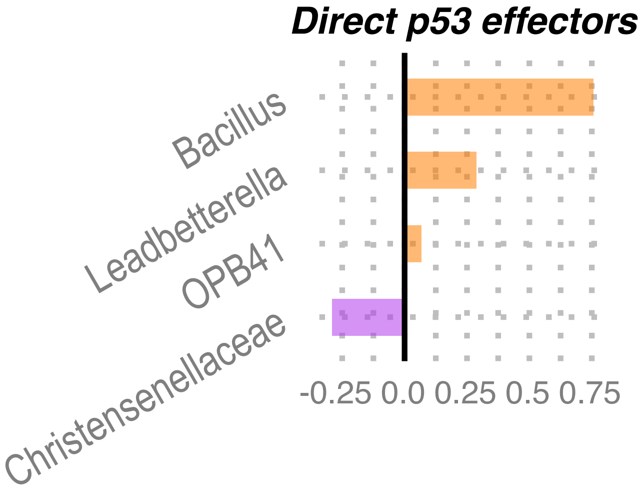

Direct p53 effectors
Bacillus
Leadbetterella
OPB41
Christensenellaceae
-0.25
0.0
0.25
0.5
0.75

ErbB1 downstream signaling

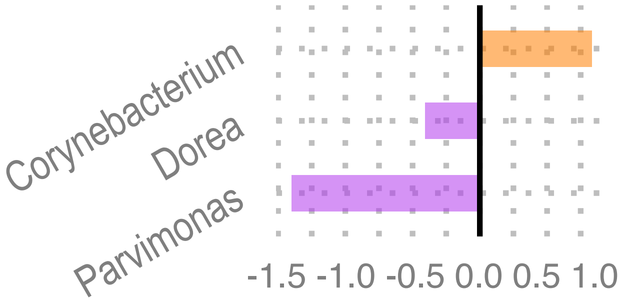

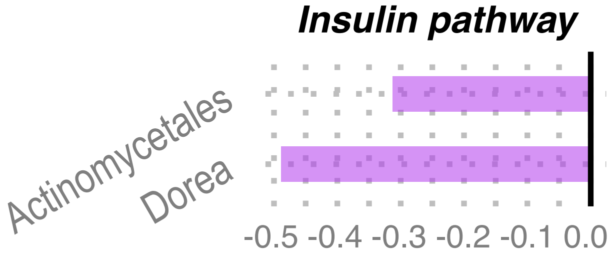
Insulin pathway
Actinomycetales
Dorea
-0.5 -0.4 -0.3 -0.2 -0.1 0.0

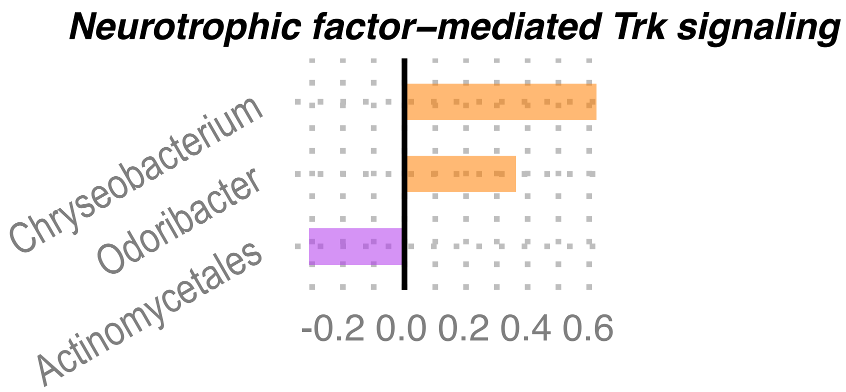
Neurotrophic factor–mediated Trk signaling
Chryseobacterium
Odoribacter
Actinomycetales
-0.2
0.0
0.2
0.4
0.6

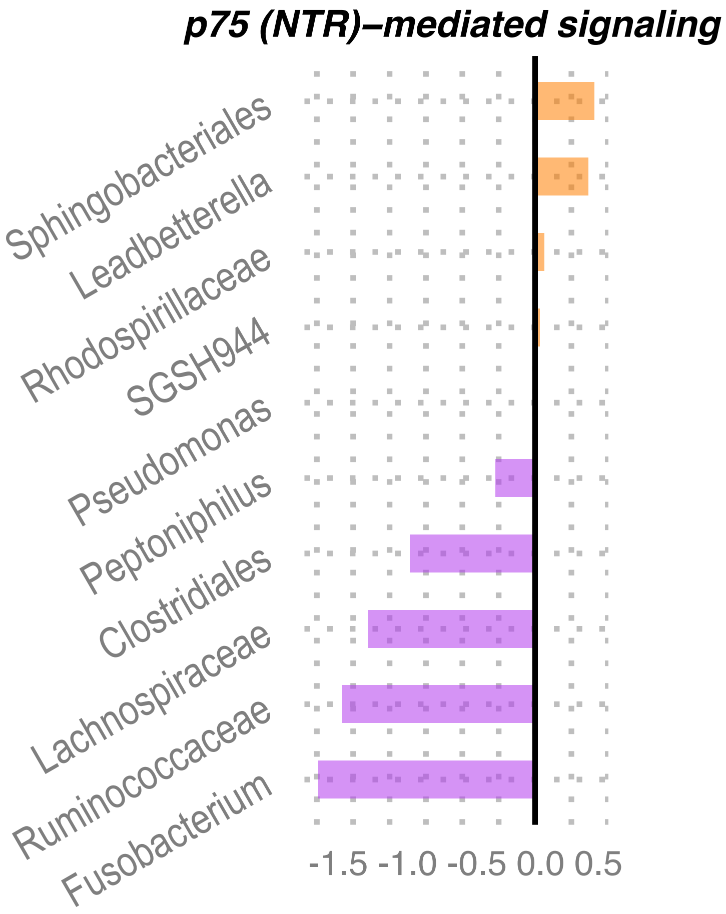

p75 (NTR)–mediated signaling
Sphingobacteriales
Leadbetterella
Rhodospirillaceae
SGSH944
Pseudomonas
Peptoniphilus
Clostridiales
Lachnospiraceae
Ruminococcaceae
Fusobacterium
-1.5
-1.0
-0.5
0.0
0.5

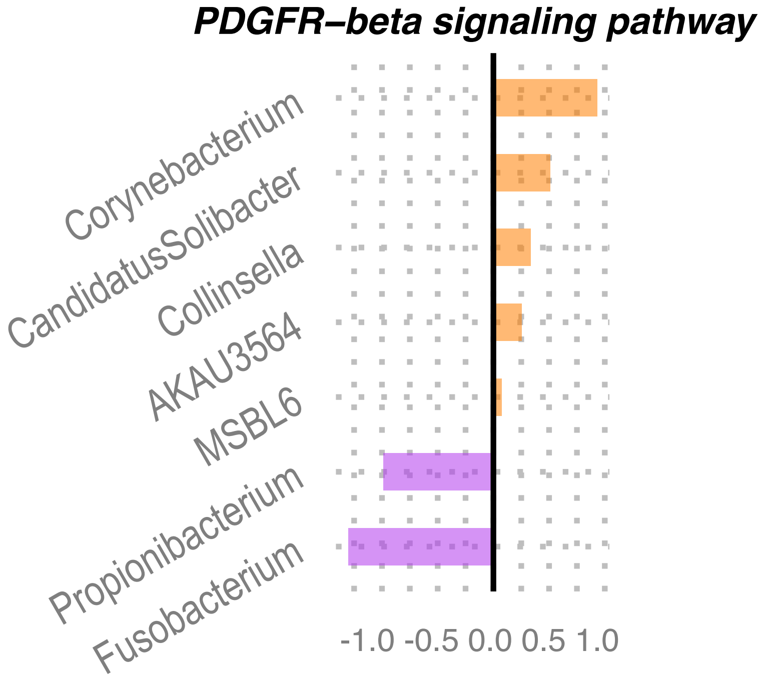
PDGFR–beta signaling pathway
Corynebacterium
CandidatusSolibacter
Collinsella
AKAU3564
MSBL6
Propionibacterium
Fusobacterium
-1.0
-0.5
0.0
0.5
1.0

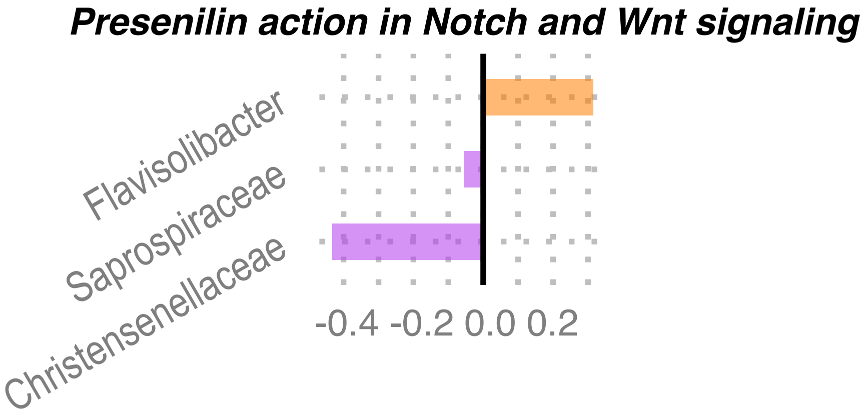

Presenilin action in Notch and Wnt signaling
Flavisolibacter
Saprospiraceae
Christensenellaceae
-0.4
-0.2
0.0
0.2

Regulation of CDC42 activity

File intentionally blank -no taxa met the abundance-difference threshold for inclusion

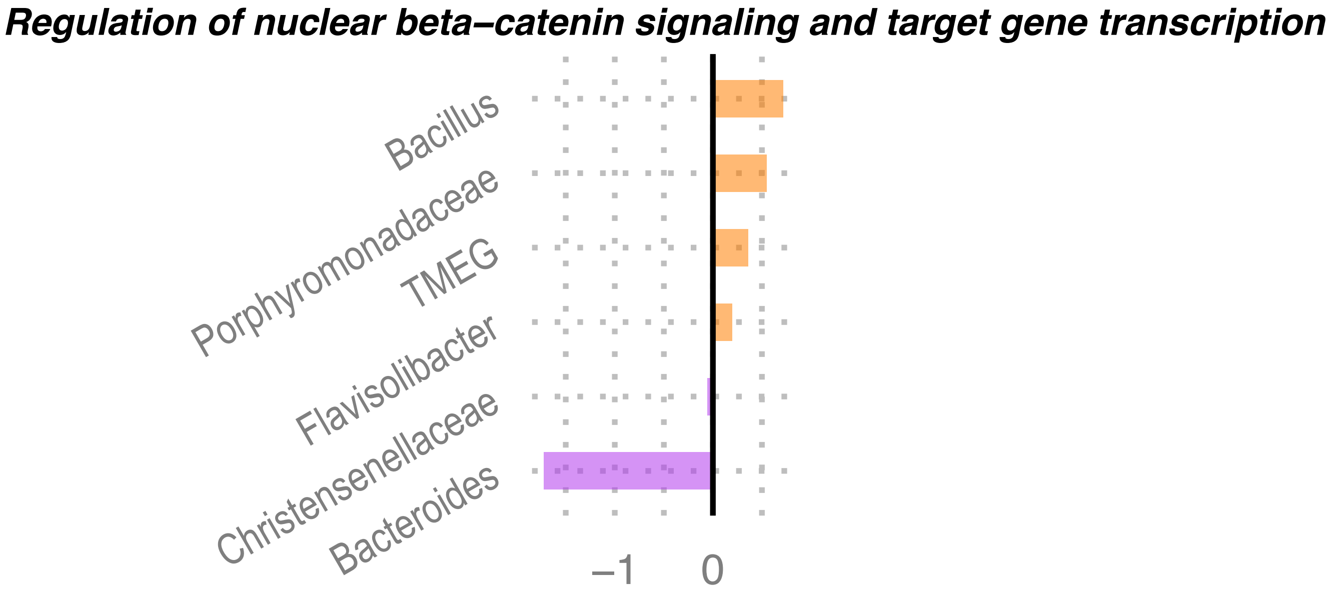
Regulation of nuclear beta−catenin signaling and target gene transcription
Bacillus
Porphyromonadaceae
TMEG
Flavisolibacter
Christensenellaceae
Bacteroides
−1
0

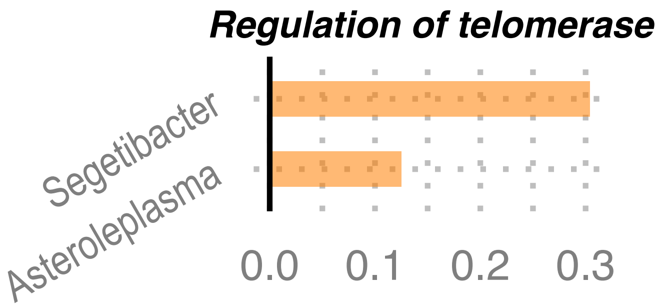

Regulation of telomerase
Segetibacter
Asteroleplasma
0.0
0.1
0.2
0.3

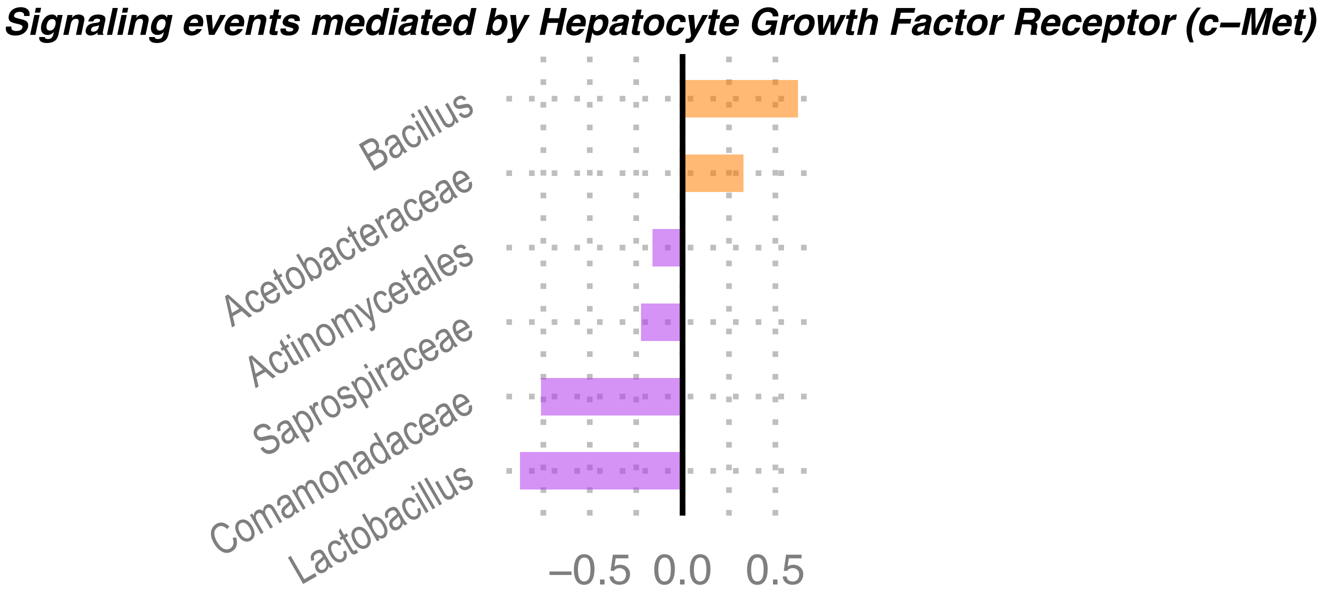
Signaling events mediated by Hepatocyte Growth Factor Receptor (c−Met)
Bacillus
Acetobacteraceae
Actinomycetales
Saprospiraceae
Comamonadaceae
Lactobacillus
−0.5
0.0
0.5

S12 Fig I Abundance Dotplots.

<u>Genes</u>

ANKRD36C

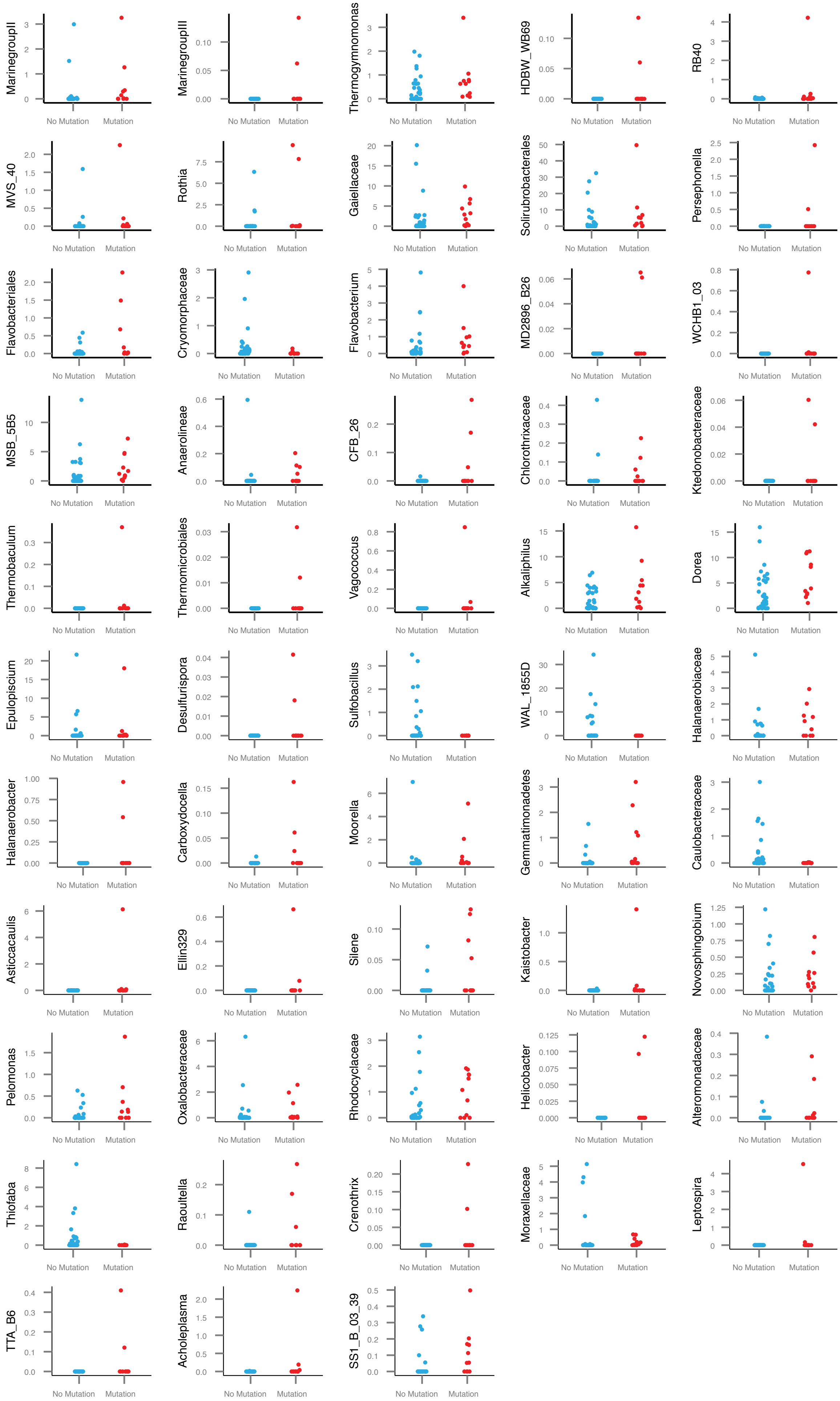
MarinegroupII
MarinegroupIII
Thermogymnomonas
HDBW_WB69
RB40
MVS_40
Rothia
Gaiellaceae
Solirubrobacterales
Persephonella
Flavobacteriales
Cryomorphaceae
Flavobacterium
MD2896_B26
WCHB1_03
MSB_5B5
Anaerolineae
CFB_26
Chlorothrixaceae
Ktedonobacteraceae
Thermobaculum
Thermomicrobiales
Vagococcus
Alkaliphilus
Dorea
Epulopiscium
Desulfurispora
Sulfobacillus
WAL_1855D
Halanaerobiaceae
Halanaerobacter
Carboxydocella
Moorella
Gemmatimonadetes
Caulobacteraceae
Asticcacaulis
Ellin329
Silene
Kaistobacter
Novosphingobium
Pelomonas
Oxalobacteraceae
Rhodocyclaceae
Helicobacter
Alteromonadaceae
Thiofaba
Raoultella
Crenothrix
Moraxellaceae
Leptospira
TTA_B6
Acholeplasma
SS1_B_03_39
No Mutation
Mutation

APC

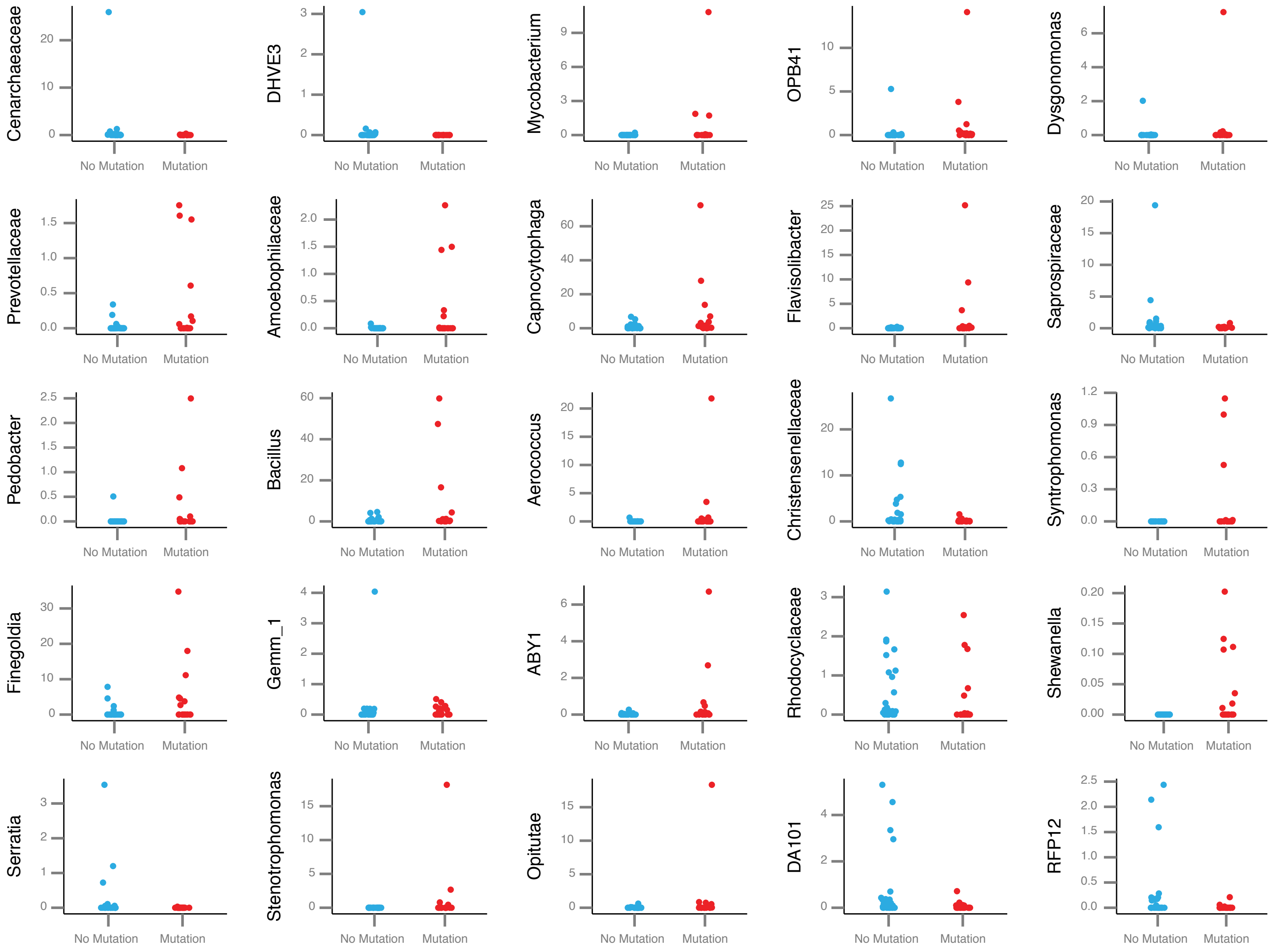

Cenarchaeaceae
DHVE3
Mycobacterium
OPB41
Dysgonomonas
Prevotellaceae
Amoebophilaceae
Capnocytophaga
Flavisolibacter
Saprospiraceae
Pedobacter
Bacillus
Aerococcus
Christensenellaceae
Syntrophomonas
Finegoldia
Gemm_1
ABY1
Rhodocyclaceae
Shewanella
Serratia
Stenotrophomonas
Opitutae
DA101
RFP12
No Mutation
Mutation

CTBP2

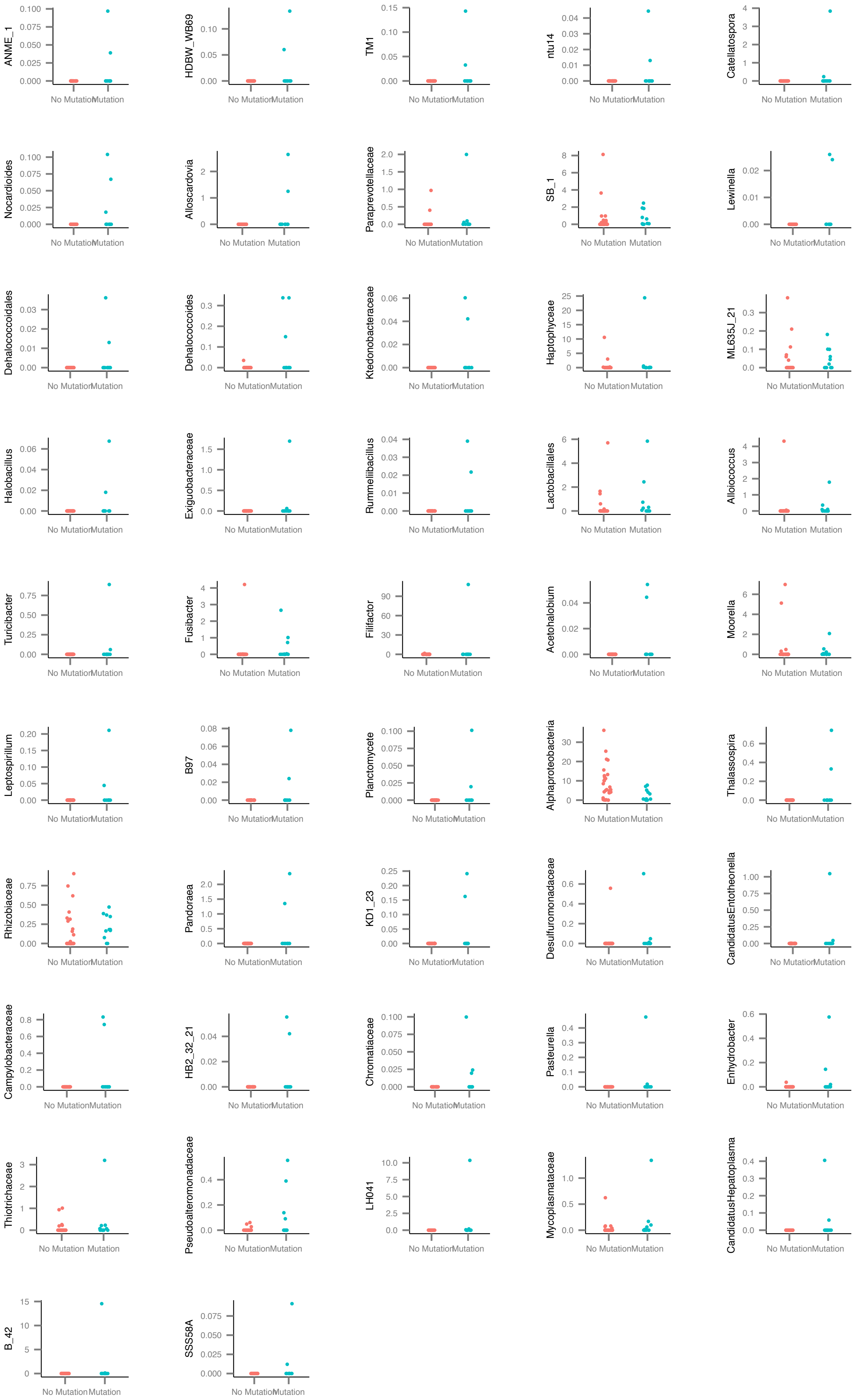
ANME_1
HDBW_WB69
TM1
ntu14
Catellatospora
Nocardioides
Alloscardovia
Paraprevotellaceae
SB_1
Lewinella
Dehalococcoidales
Dehalococcoides
Ktedonobacteraceae
Haptophyceae
ML635J_21
Halobacillus
Exiguobacteraceae
Rummeliibacillus
Lactobacillales
Alloiococcus
Turicibacter
Fusibacter
Filifactor
Acetohalobium
Moorella
Leptospirillum
B97
Planctomycete
Alphaproteobacteria
Thalassospira
Rhizobiaceae
Pandoraea
KD1_23
Desulfuromonadaceae
CandidatusEntotheonella
Campylobacteraceae
HB2_32_21
Chromatiaceae
Pasteurella
Enhydrobacter
Thiotrichaceae
Pseudoalteromonadaceae
LH041
Mycoplasmataceae
CandidatusHepatoplasma
B_42
SS58A
No Mutation
Mutation

KMT2C

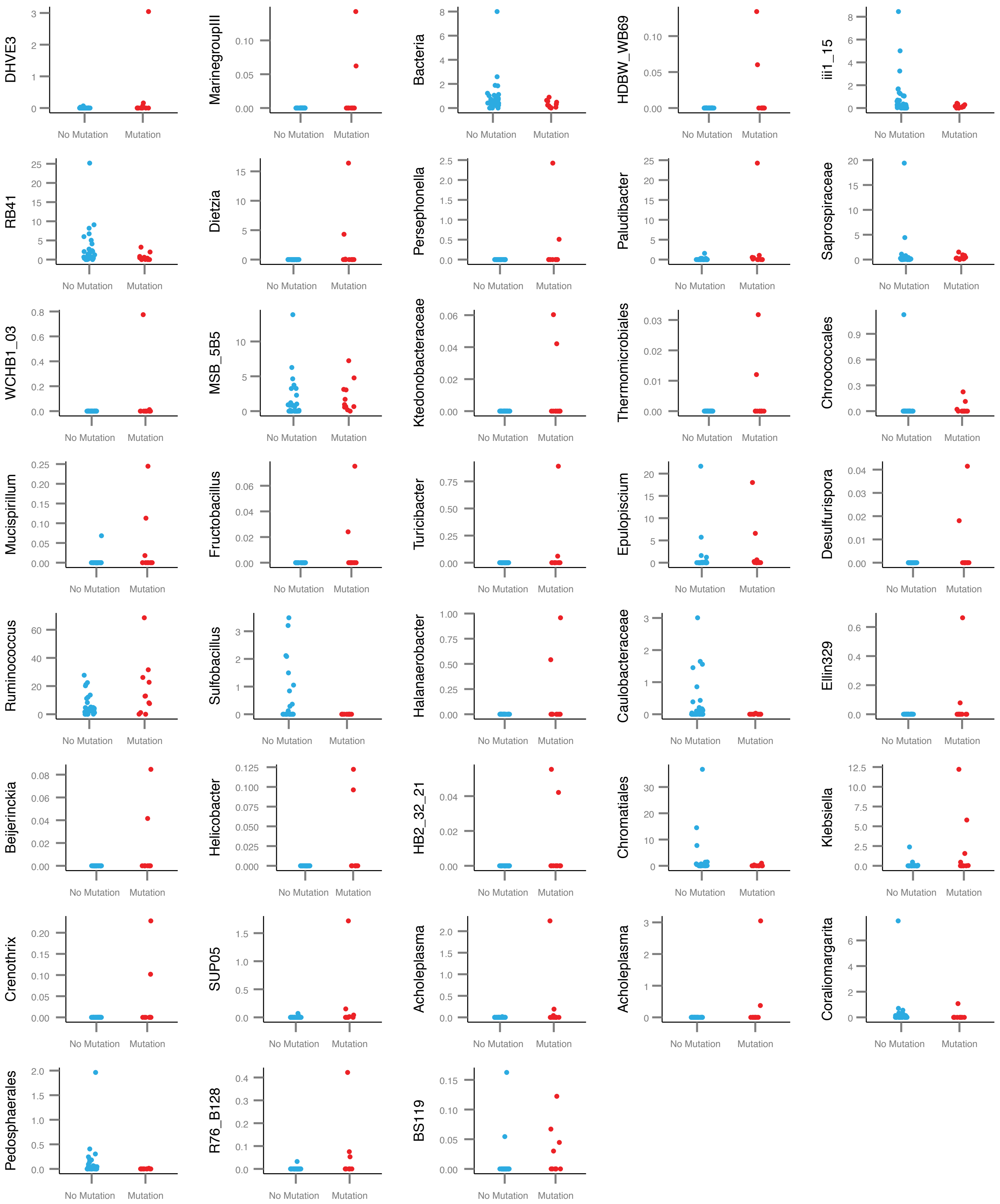
DHVE3
MarinegroupIII
Bacteria
HDBW_WB69
iii1_15
RB41
Dietzia
Persephonella
Paludibacter
Saprospiraceae
WCHB1_03
MSB_5B5
Ktedonobacteraceae
Thermomicrobiales
Chroococcales
Mucispirillum
Fructobacillus
Turicibacter
Epulopiscium
Desulfurispora
Ruminococcus
Sulfobacillus
Halanaerobacter
Caulobacteraceae
Ellin329
Beijerinckia
Helicobacter
HB2_32_21
Chromatiales
Klebsiella
Crenothrix
SUP05
Acholeplasma
Acholeplasma
Coraliomargarita
Pedosphaerales
R76_B128
BS119
No Mutation
Mutation

ZNF717

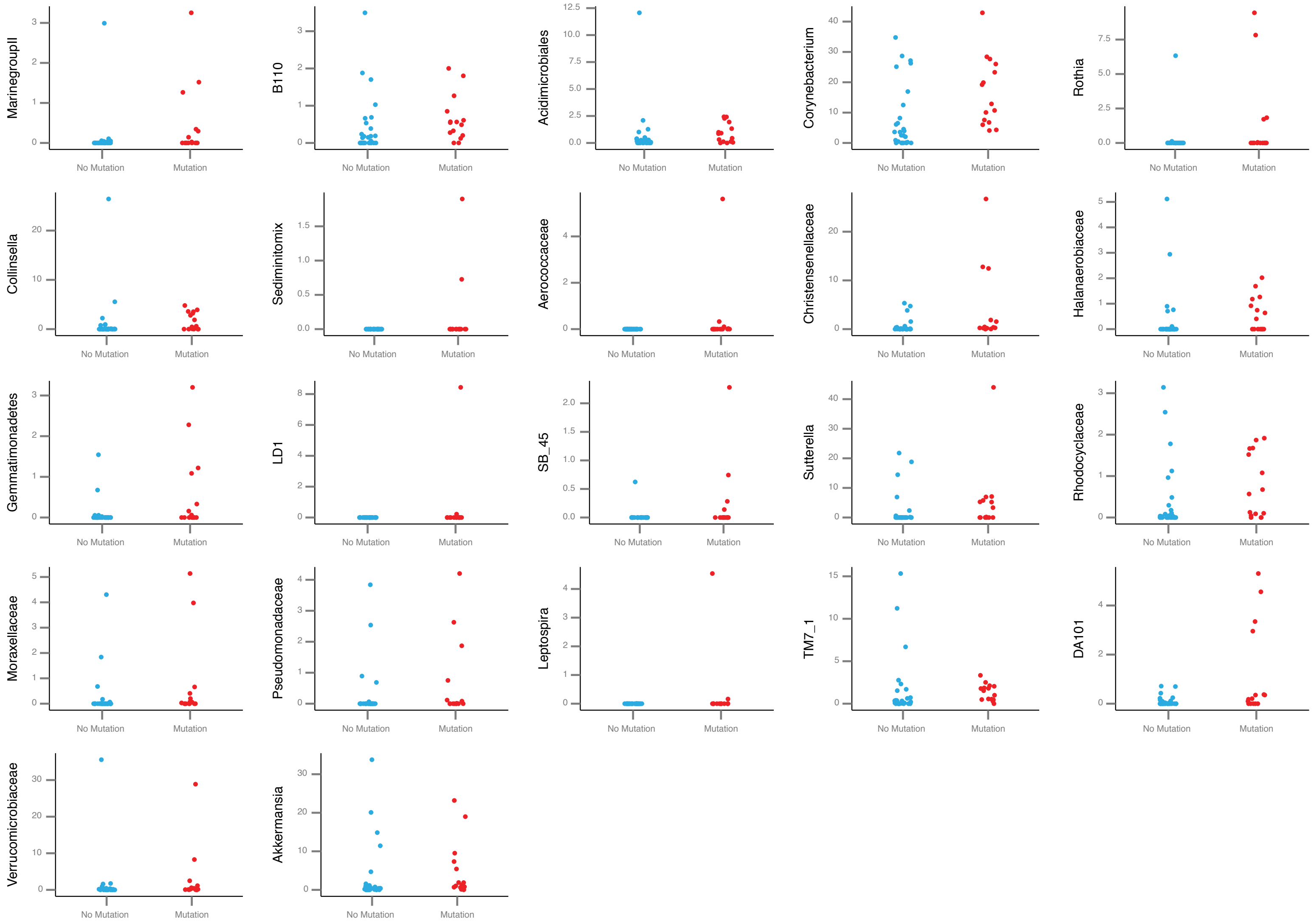
MarinegroupII
B110
Acidimicrobiales
Corynebacterium
Rothia
Collinsella
Sediminitomix
Aerococcaceae
Christensenellaceae
Halanaerobiaceae
Gemmatimonadetes
LD1
SB_45
Sutterella
Rhodocyclaceae
Moraxellaceae
Pseudomonadaceae
Leptospira
TM7_1
DA101
Verrucomicrobiaceae
Akkermansia
No Mutation
Mutation

S12 Fig | Abundance Dotplots.

KEGG

ABC Transporters

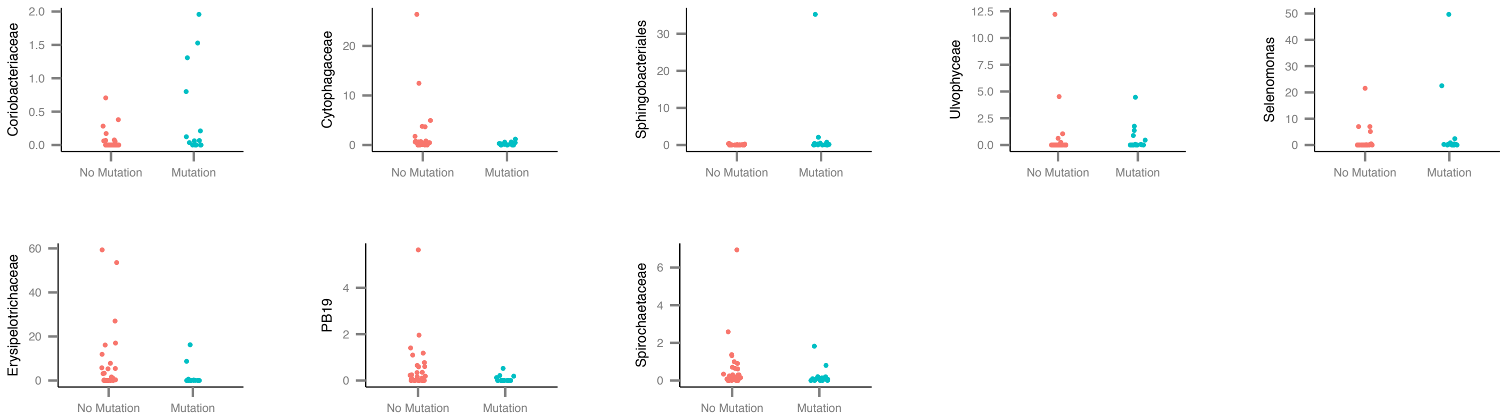

Adherens junctions

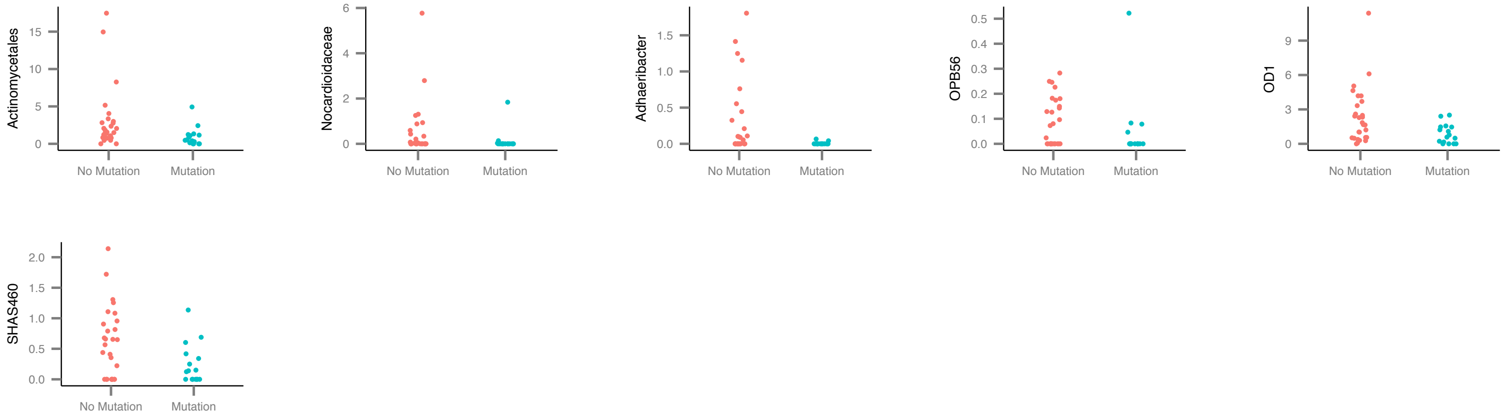

Alanine, aspartate, and glutamate metabolism

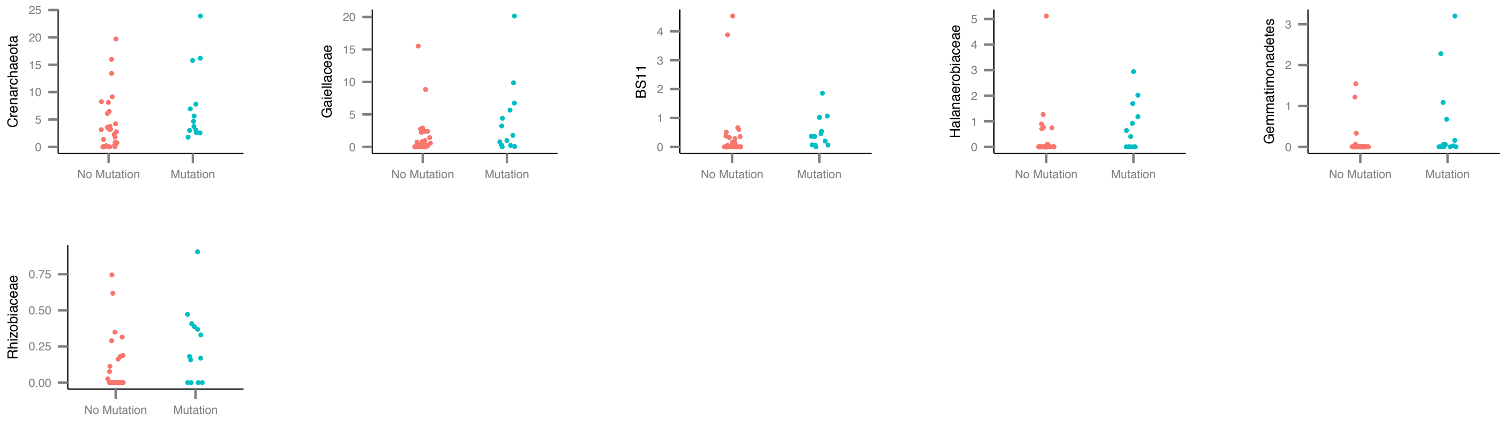

Cell cycle

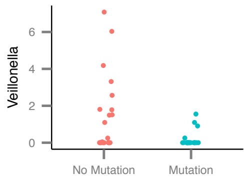

Veillonella
6
4
2
0
No Mutation
Mutation

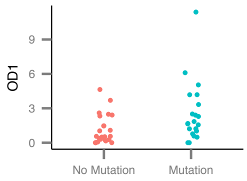

OD1
9
6
3
0
No Mutation
Mutation

Cysteine and methionine metabolism

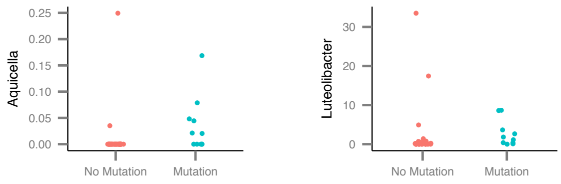

Focal adhesion

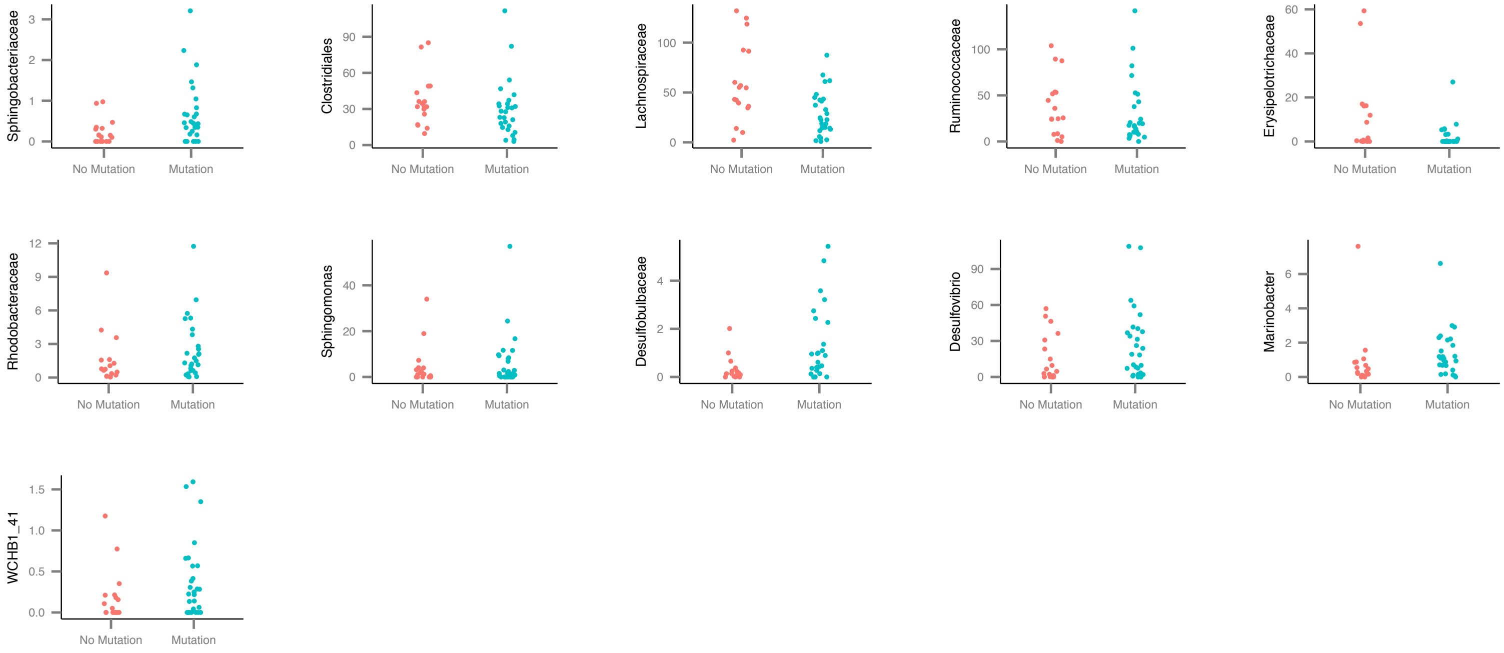

Lysine degradation

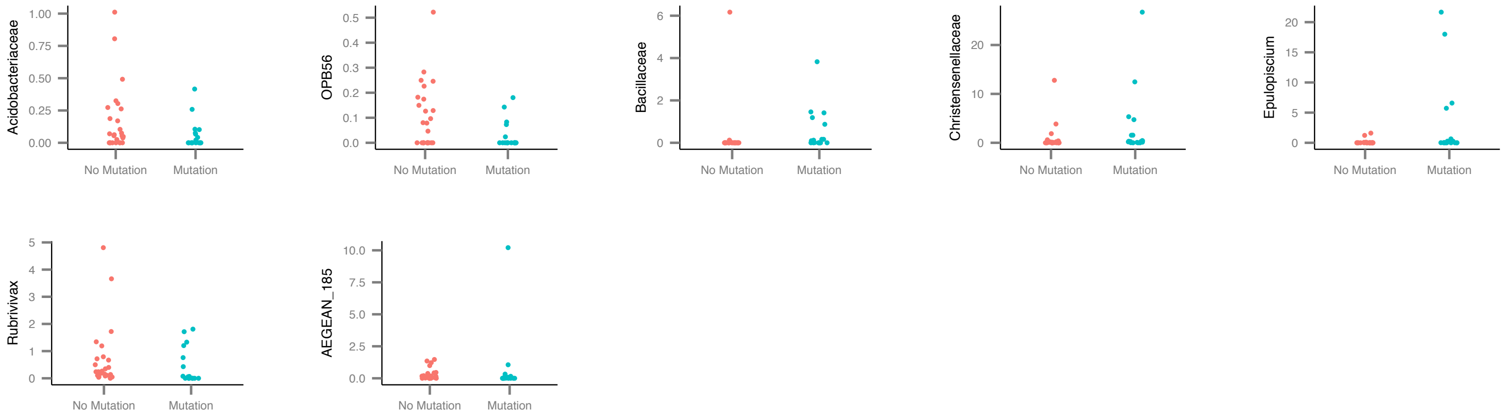

MAPK signaling pathway

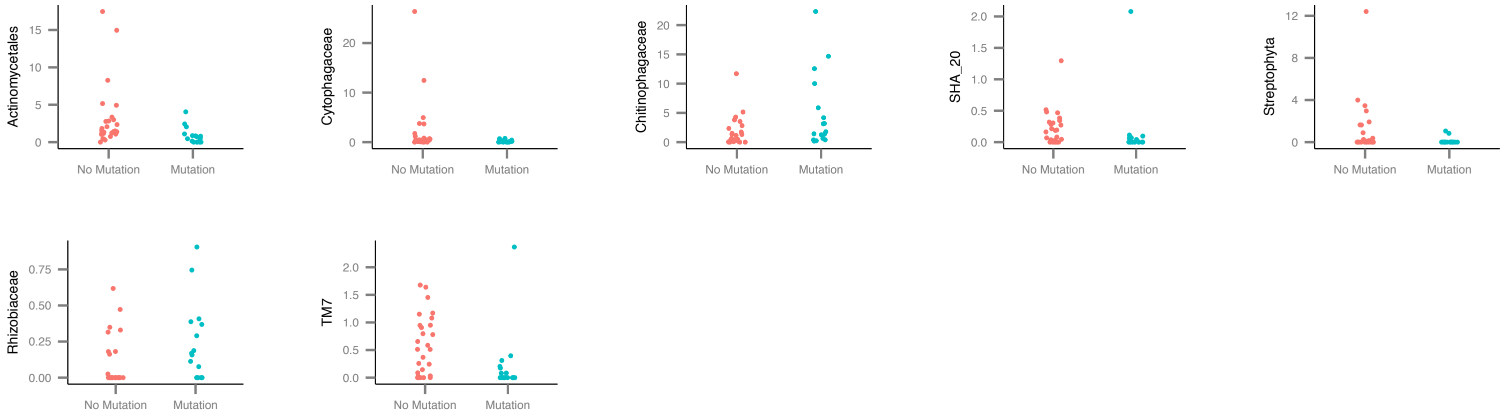

Notch signaling pathway

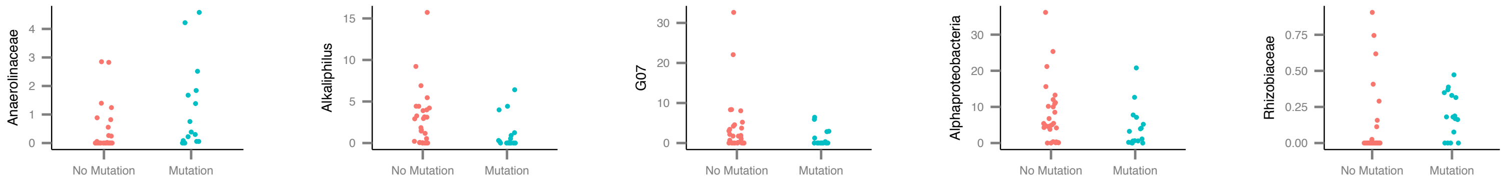

Oocyte meiosis

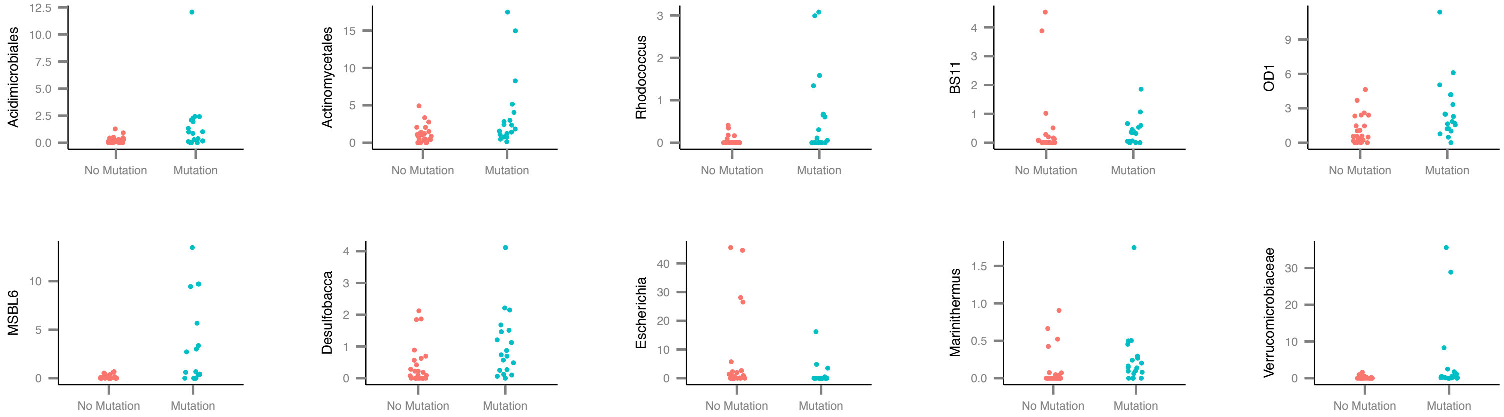

Oxidative phosphorylation

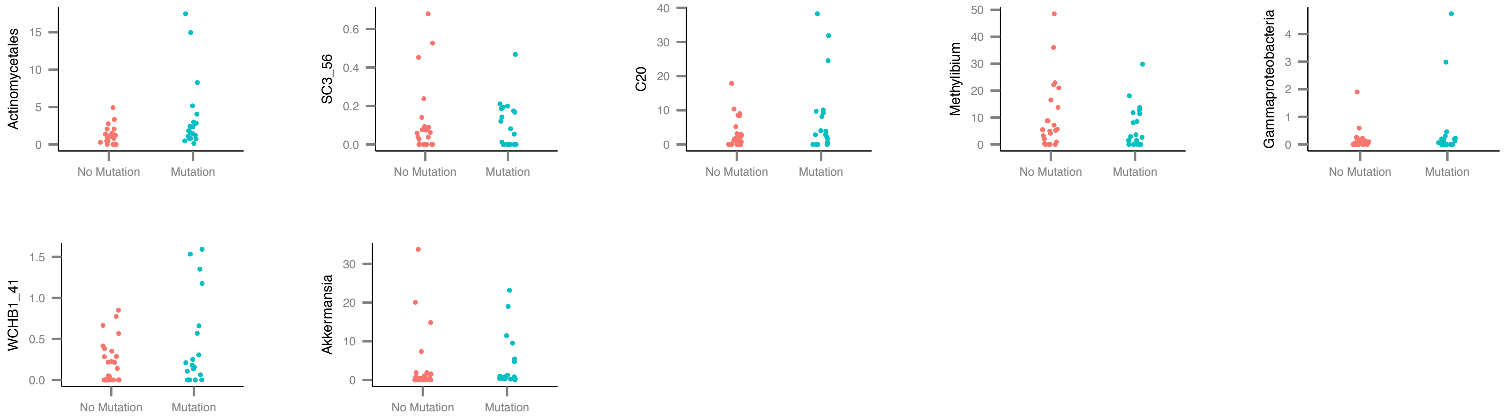

Phosphatidylinositol signaling system

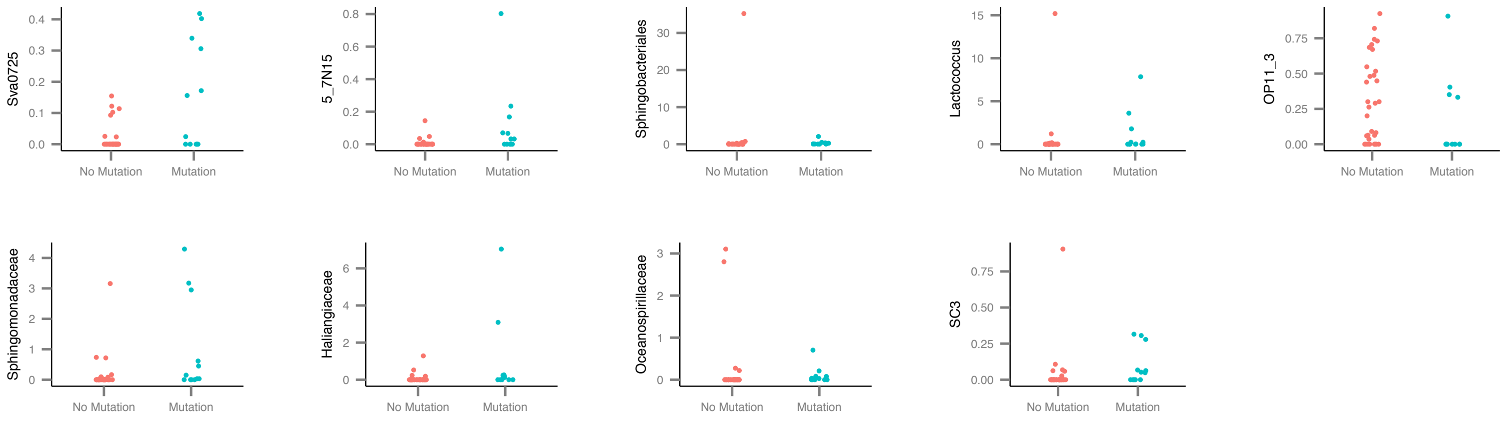

Protein processing in endoplasmic reticulum

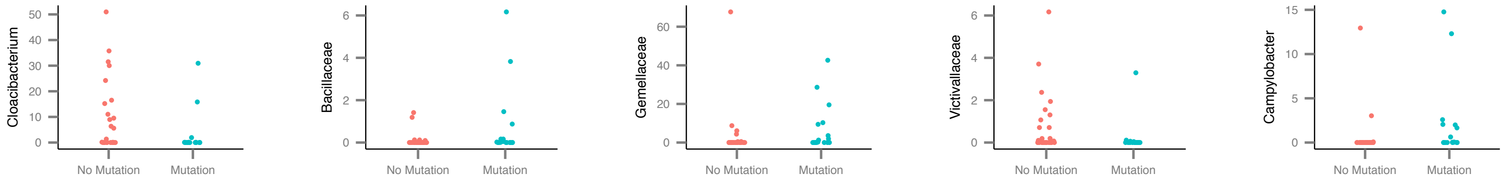

Purine metabolism

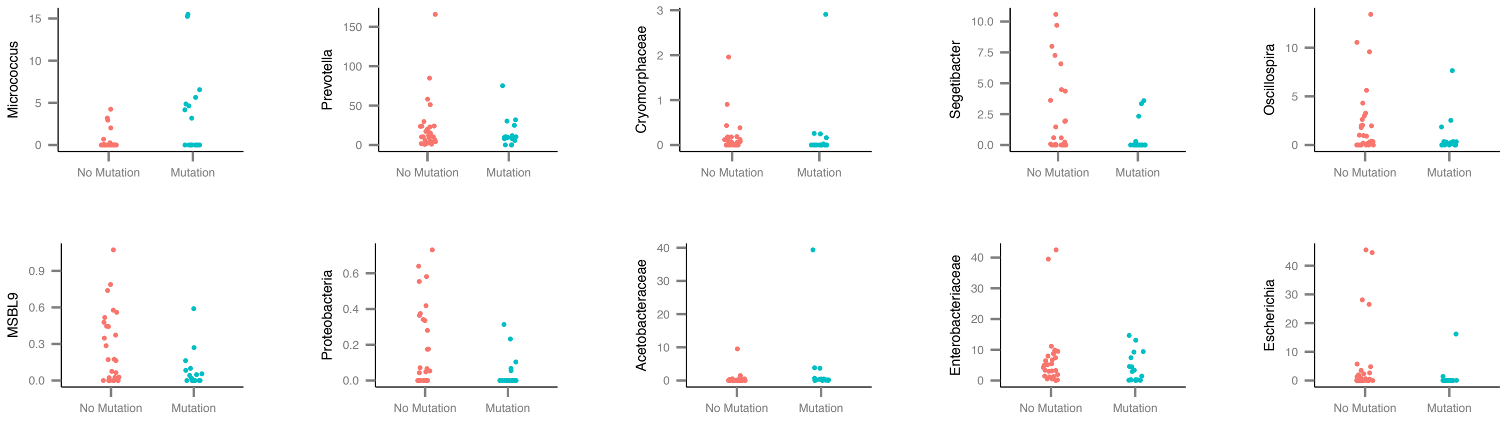

Regulation of actin cytoskeleton

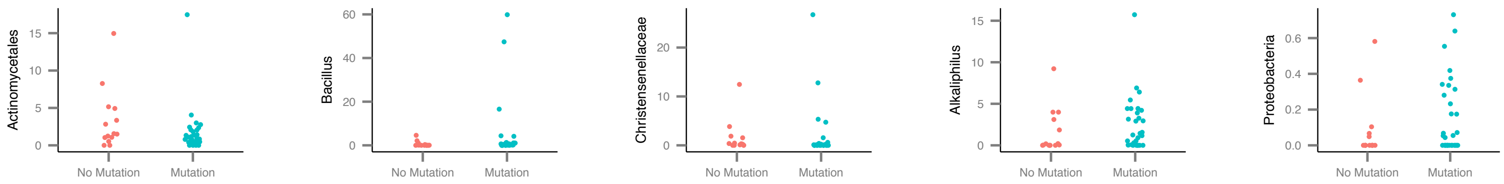

Ribosome

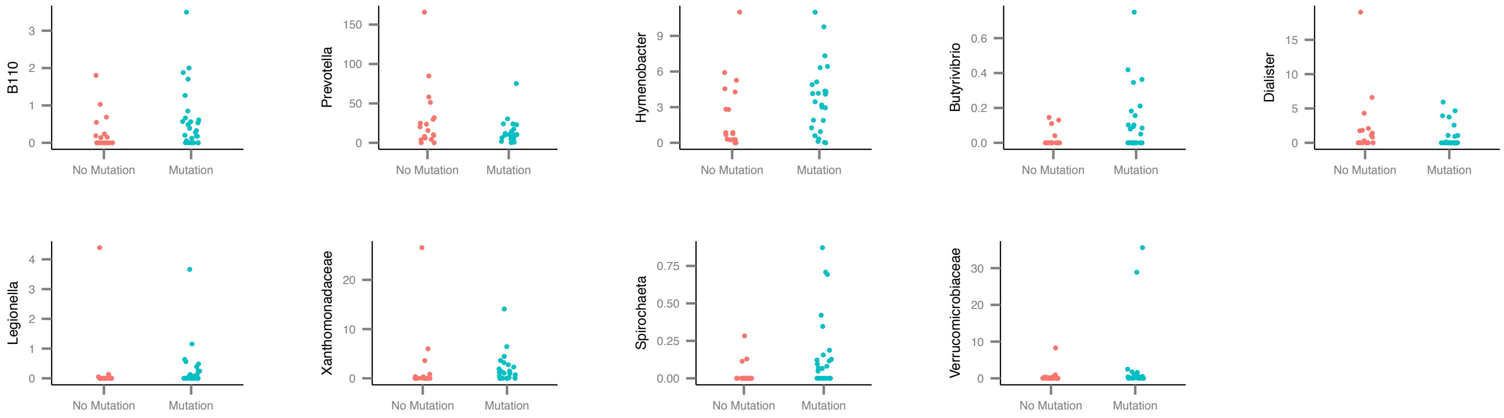
B110
Prevotella
Hymenobacter
Butyrivibrio
Dialister
Legionella
Xanthomonadaceae
Spirochaeta
Verrucomicrobiaceae
No Mutation
Mutation

RNA degradation

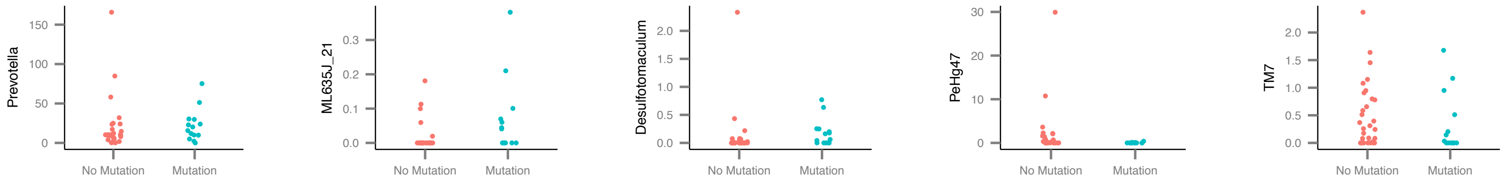

RNA transport

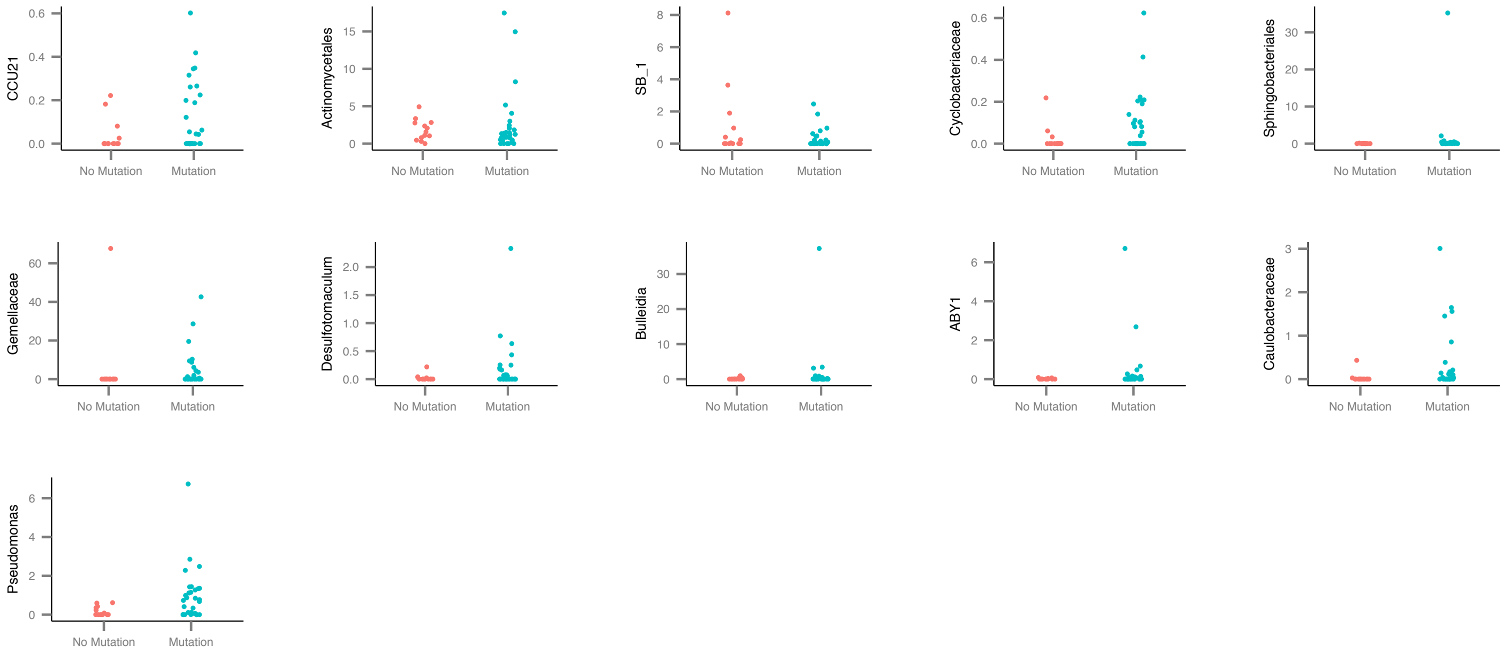

Spliceosome

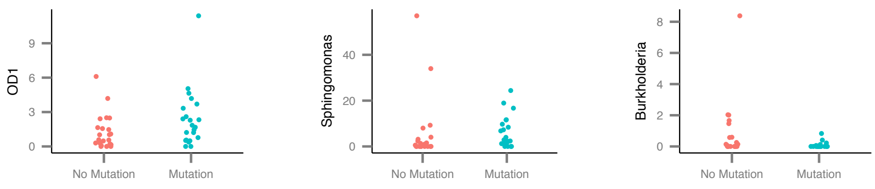

Ubiquitin mediated proteolysis

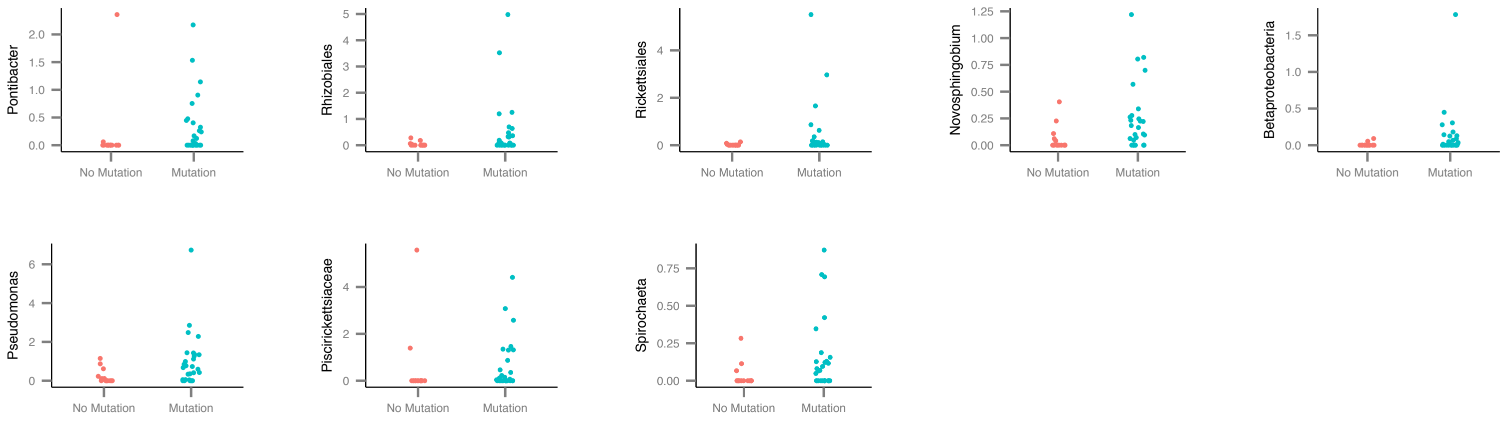

Wnt signaling pathway

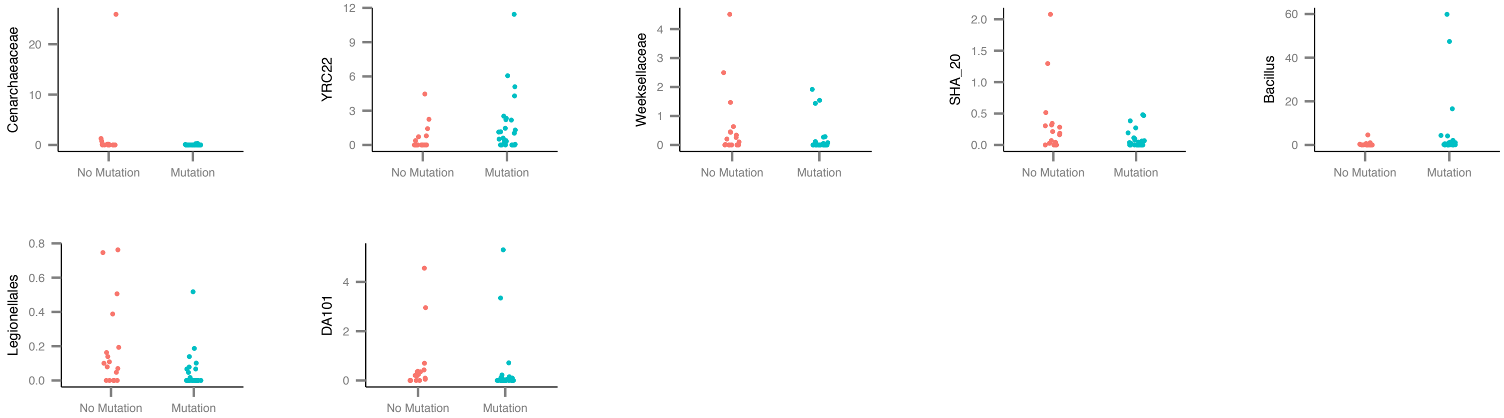

S12 Fig | Abundance Dotplots.

PID

Canonical Wnt signaling pathway

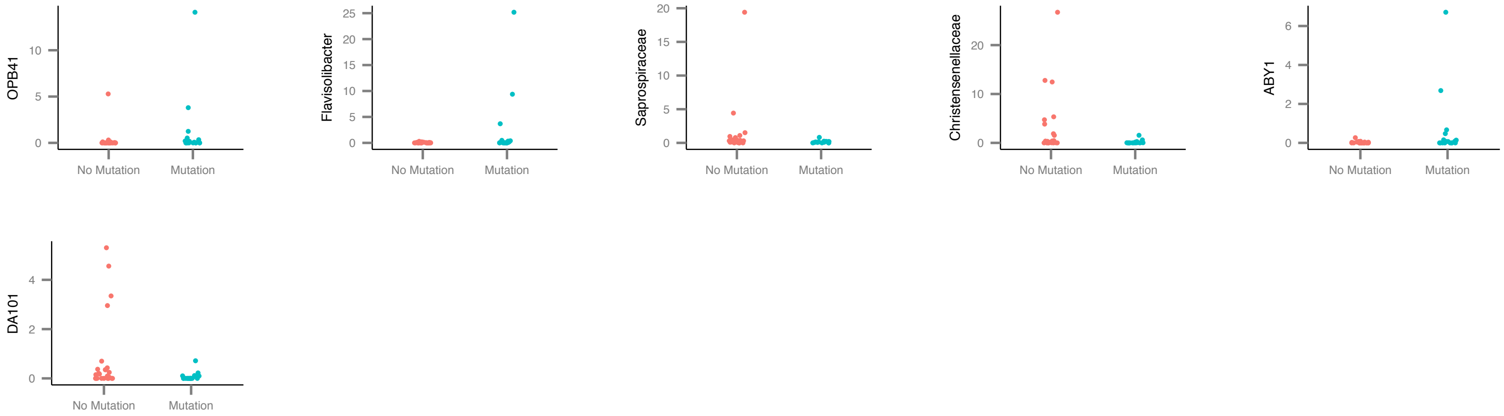

CDC42 signaling

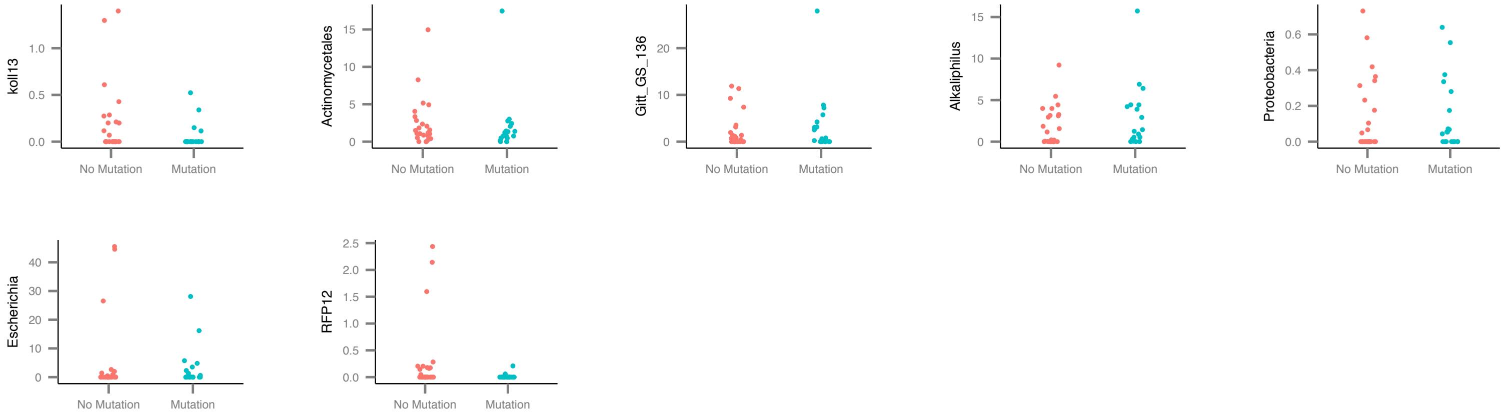

CXCR4-mediated signaling

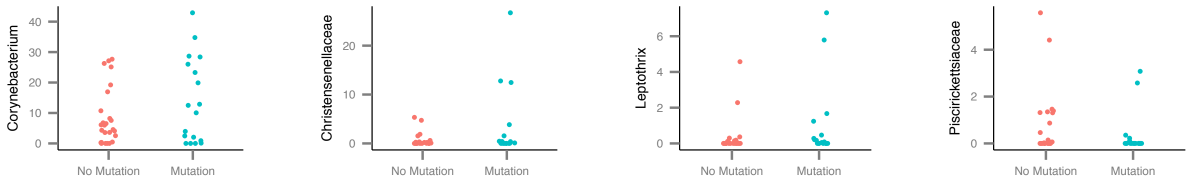

Degradation of beta-catenin

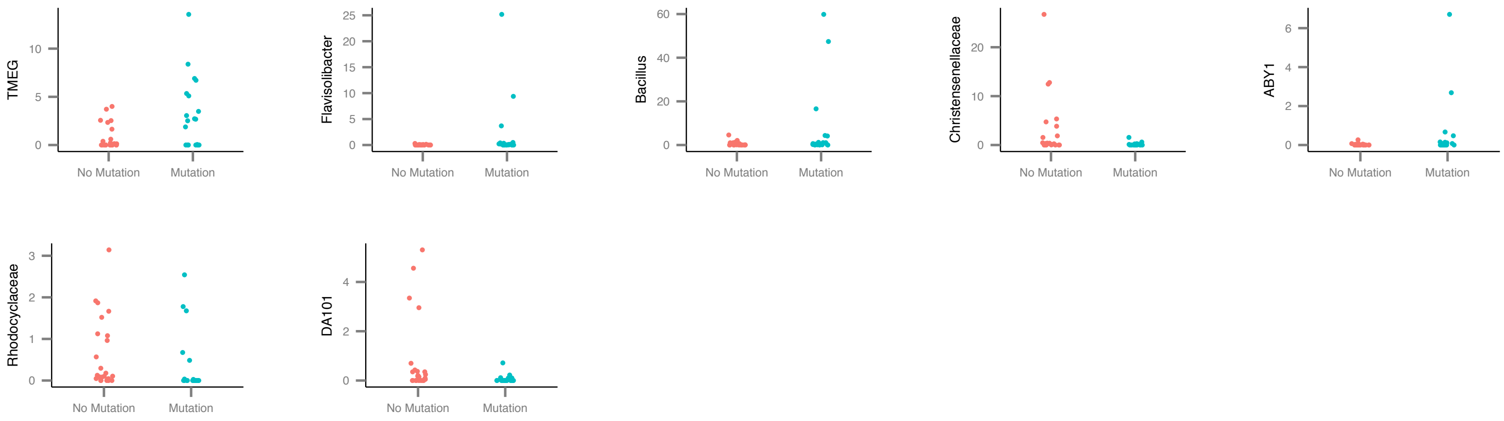

Direct p53 effectors

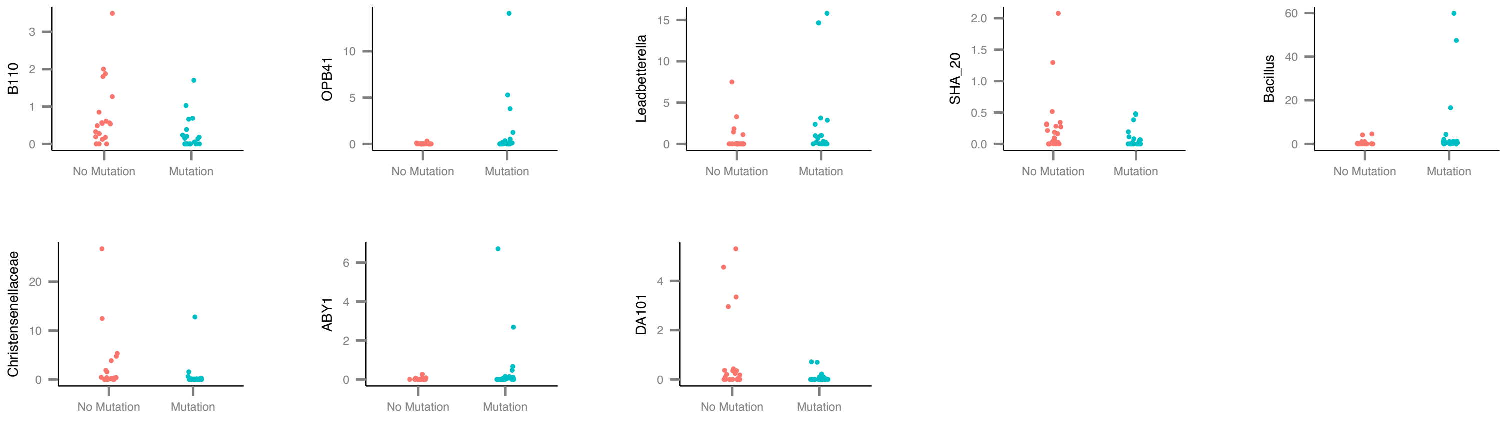

ErbB1 downstream signaling

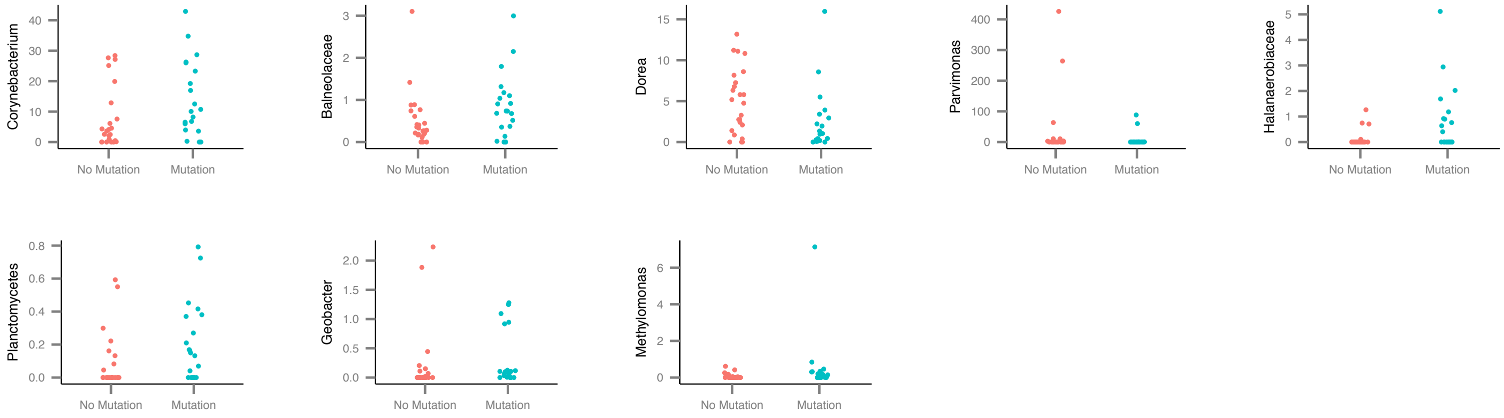

Insulin pathway

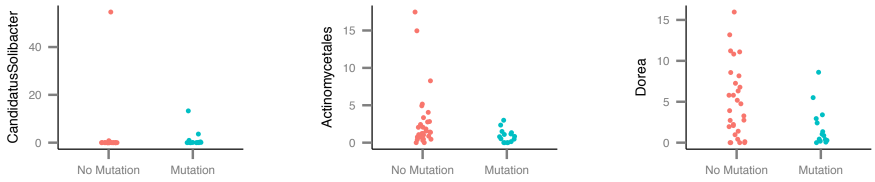

Neurotrophic factor-mediated Trk signaling

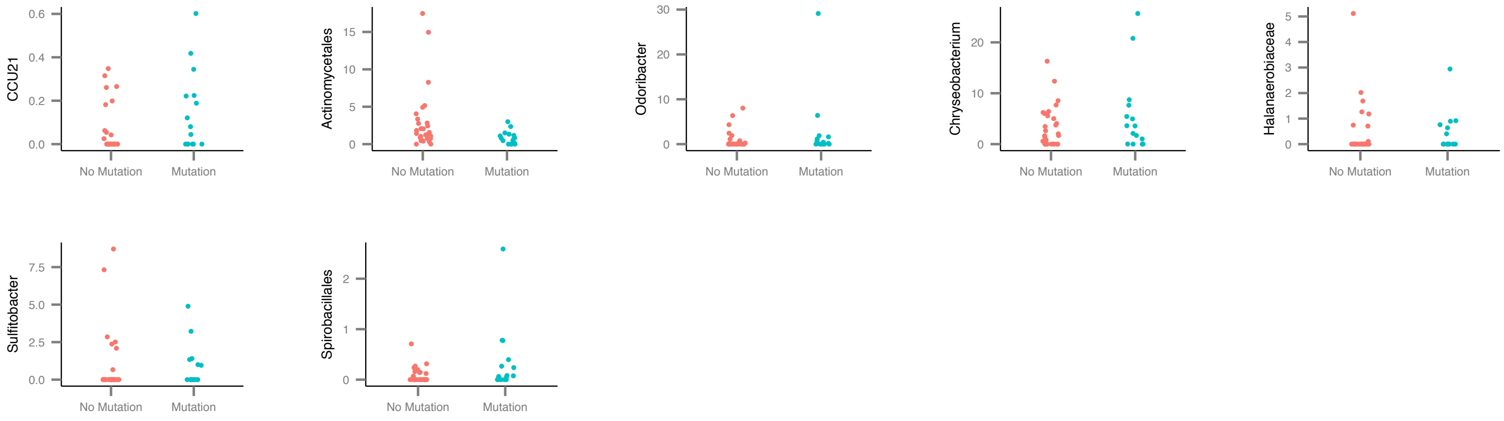

p75 (NTR)-mediated signaling

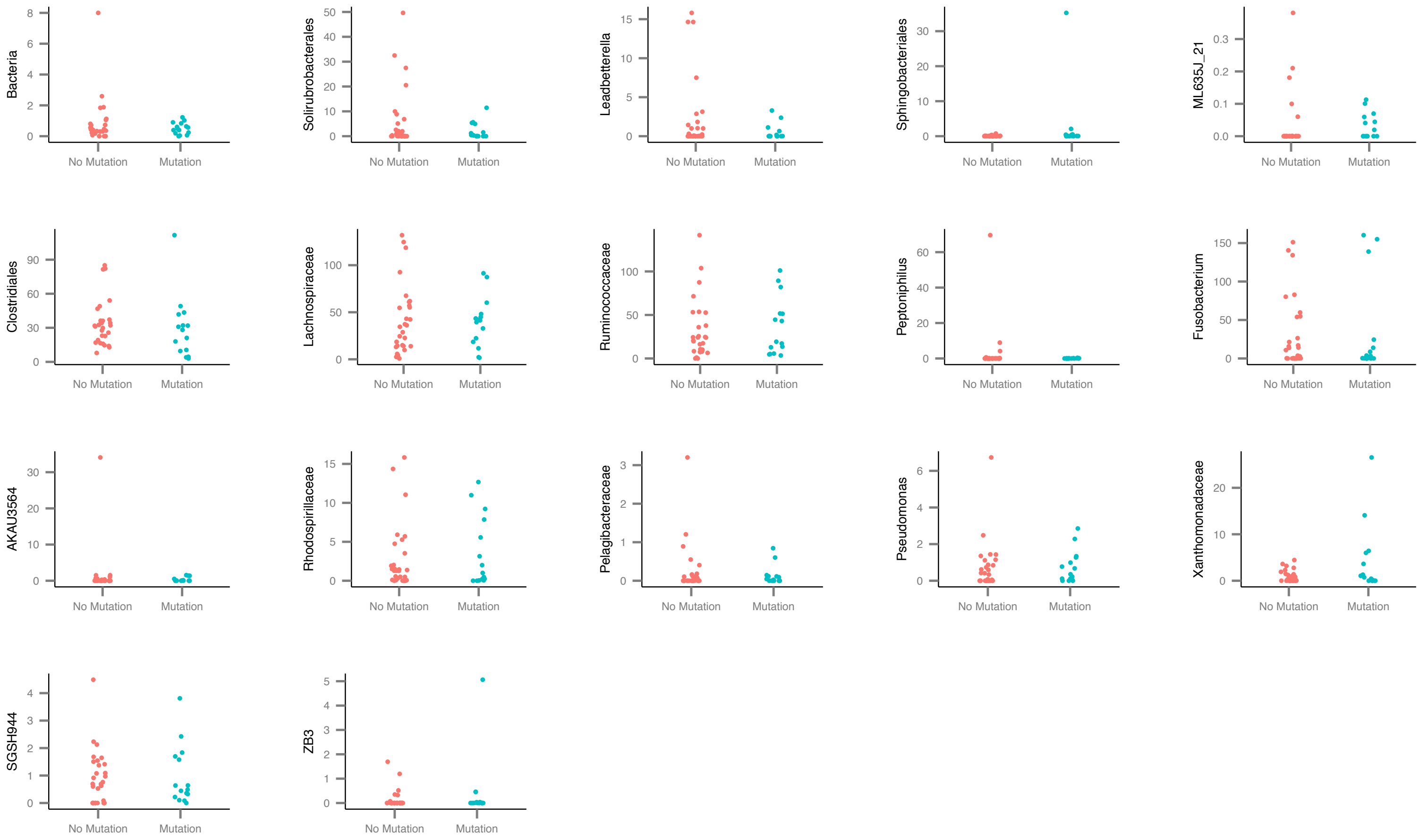

PDGFR-beta signaling pathway

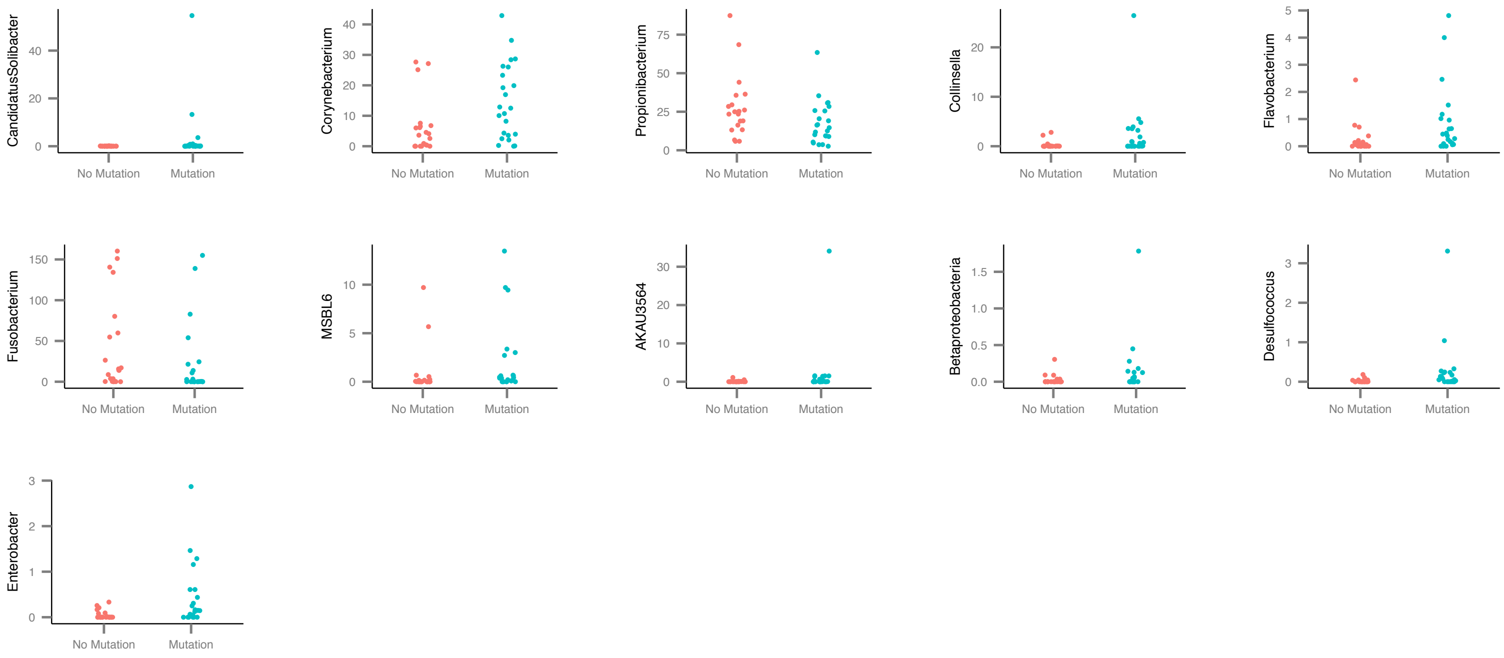

Presenilin action in Notch and Wnt signaling

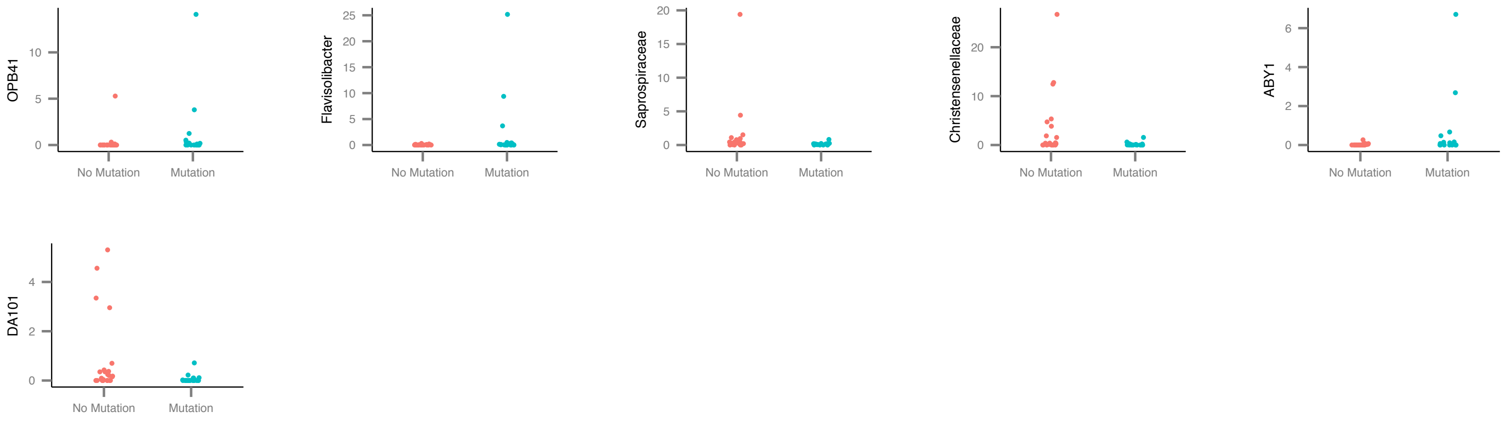

Regulation of CDC42 activity

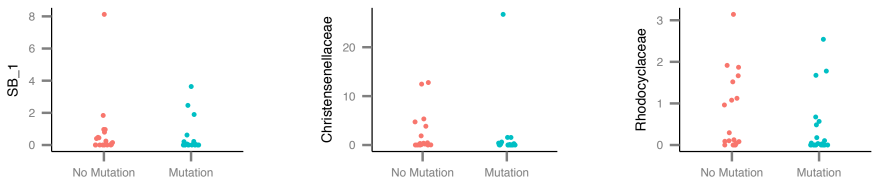

Regulation of nuclear beta-catenin signaling and target gene transcription

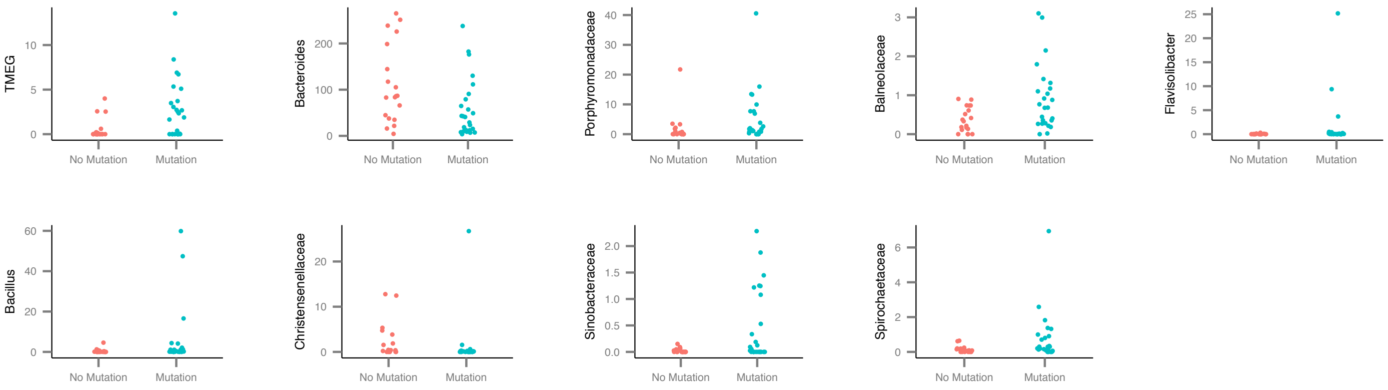

Regulation of telomerase

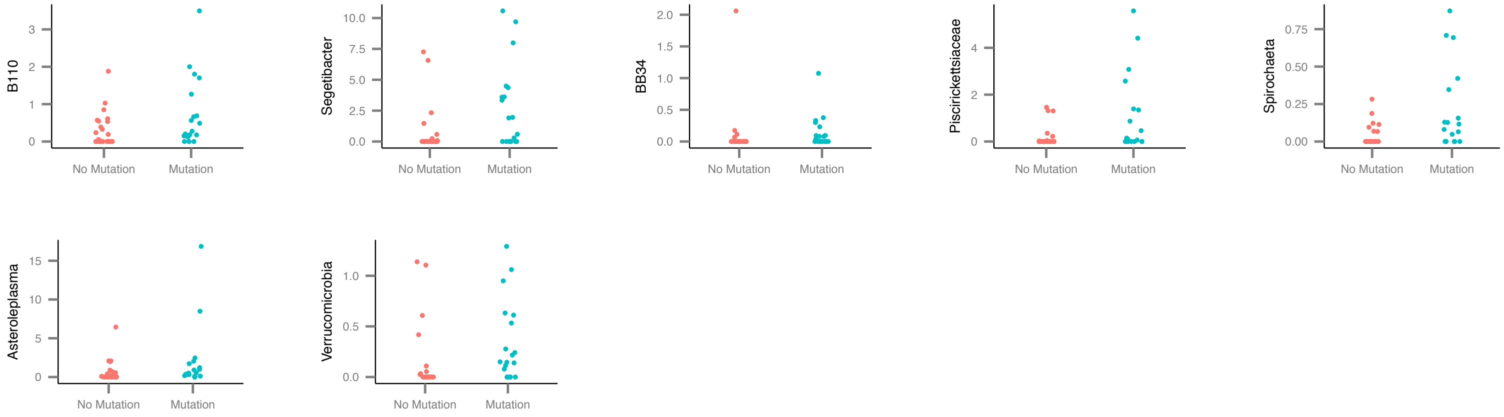

Signaling events mediated by Hepatocyte Growth Factor Receptor (c-Met)

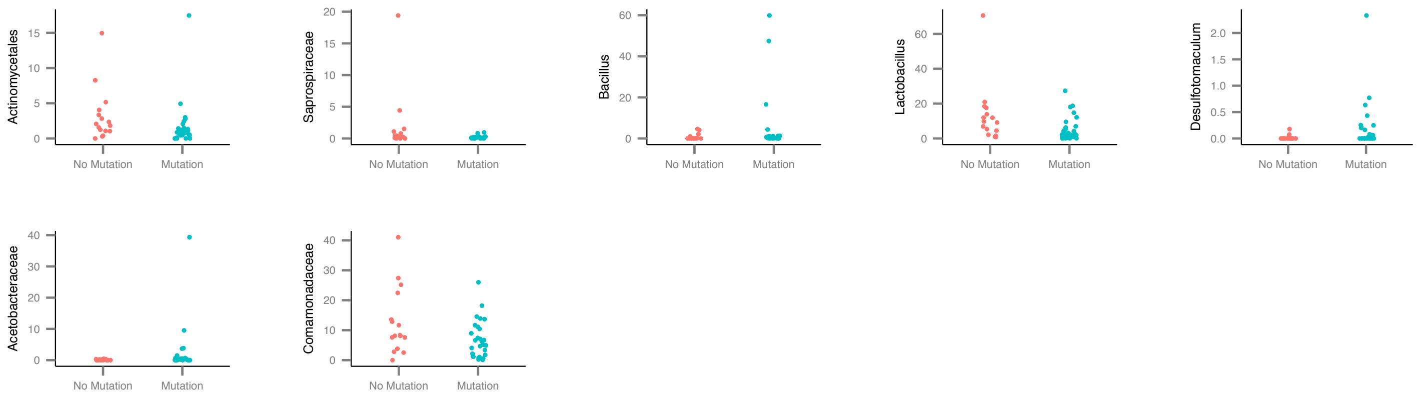

Supplement: S12 Fig — Abundances are plotted as both column dot plots as well as horizontal bar plots of the differences in the mean abundances of a subset of the taxa predicted to interact differentially with tumors with a LoF mutation relative to those without the indicated mutation. These subsets represent those taxa that had a mean difference in abundance of greater than 0.1%, proportionally. (PDF) [file pgen.1007376.s028.pdf]
