# Supplementary material for: Colorectal cancer mutational profiles correlate with defined microbial communities in the tumor microenvironment
Source: PLoS Genet. 2018 Jun 20;14(6):e1007376. doi: 10.1371/journal.pgen.1007376 (PMC6028121; doi:10.1371/journal.pgen.1007376)
Supplement: S13 Fig — A density histogram showing the distribution of prediction accuracies for individual genes (red), KEGG pathways (green), and PID pathways (blue). Interaction prediction accuracies are highest for human cancer-specific pathways from PID. The genes (red) exhibit a double peak distribution due to the relatively high accuracy achieved when predicting the presence of LoF mutations in KMT2C (80%). (PDF) [file pgen.1007376.s029.pdf]

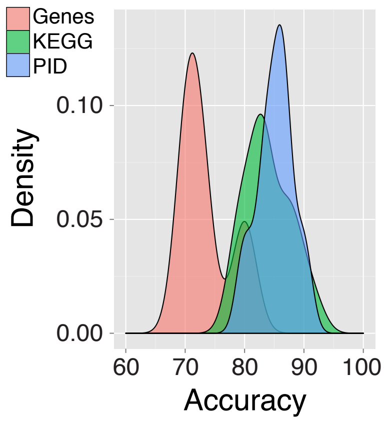

S13 Fig. Interaction prediction accuracies increase when assessing biological pathways. A density histogram showing the distribution of prediction accuracies for individual genes (red), KEGG pathways (green), and PID pathways (blue). Interaction prediction accuracies are highest for human cancer-specific pathways from PID. The genes (red) exhibit a double peak distribution due to the relatively high accuracy achieved when predicting the presence of LoF mutations in KMT2C (80%).
