# Supplementary material for: Colorectal cancer mutational profiles correlate with defined microbial communities in the tumor microenvironment
Source: PLoS Genet. 2018 Jun 20;14(6):e1007376. doi: 10.1371/journal.pgen.1007376 (PMC6028121; doi:10.1371/journal.pgen.1007376)
Supplement: S14 Fig — Horizontal bar chart indicates the total numbers of genes within each of the 4 PID pathways. Vertical bars in the bar chart indicate numbers of genes that fall within the memberships indicated by filled in dots and lines at the intersections below the bars. (PDF) [file pgen.1007376.s030.pdf]

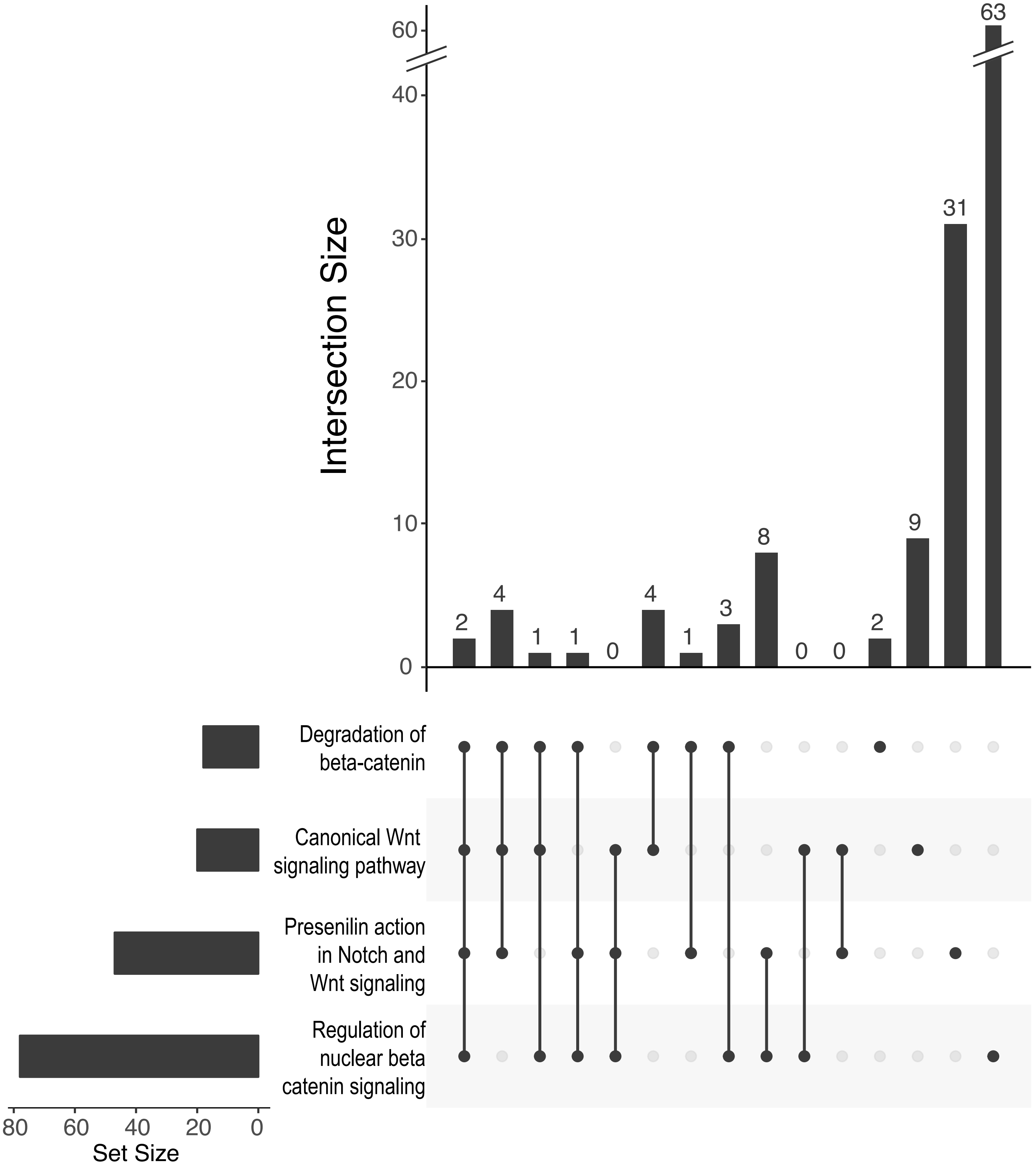

S14 Fig. Plot demonstrating the co-membership of genes within PID pathways. Horizontal bar chart indicates the total numbers of genes within each of the 4 PID pathways. Vertical bars in the bar chart indicate numbers of genes that fall within the memberships indicated by filled in dots and lines at the intersections below the bars.
