# Supplementary material for: Colorectal cancer mutational profiles correlate with defined microbial communities in the tumor microenvironment
Source: PLoS Genet. 2018 Jun 20;14(6):e1007376. doi: 10.1371/journal.pgen.1007376 (PMC6028121; doi:10.1371/journal.pgen.1007376)
Supplement: S5 Fig — (A) Scaled taxon abundances (columns) in the tumor samples that harbor LoF mutations in the genes indicated (rows). (B) Scaled differences (tumor abundance—matched normal abundance) patients that harbor tumor- specific LoF mutations in the genes indicated (rows). (PDF) [file pgen.1007376.s021.pdf]

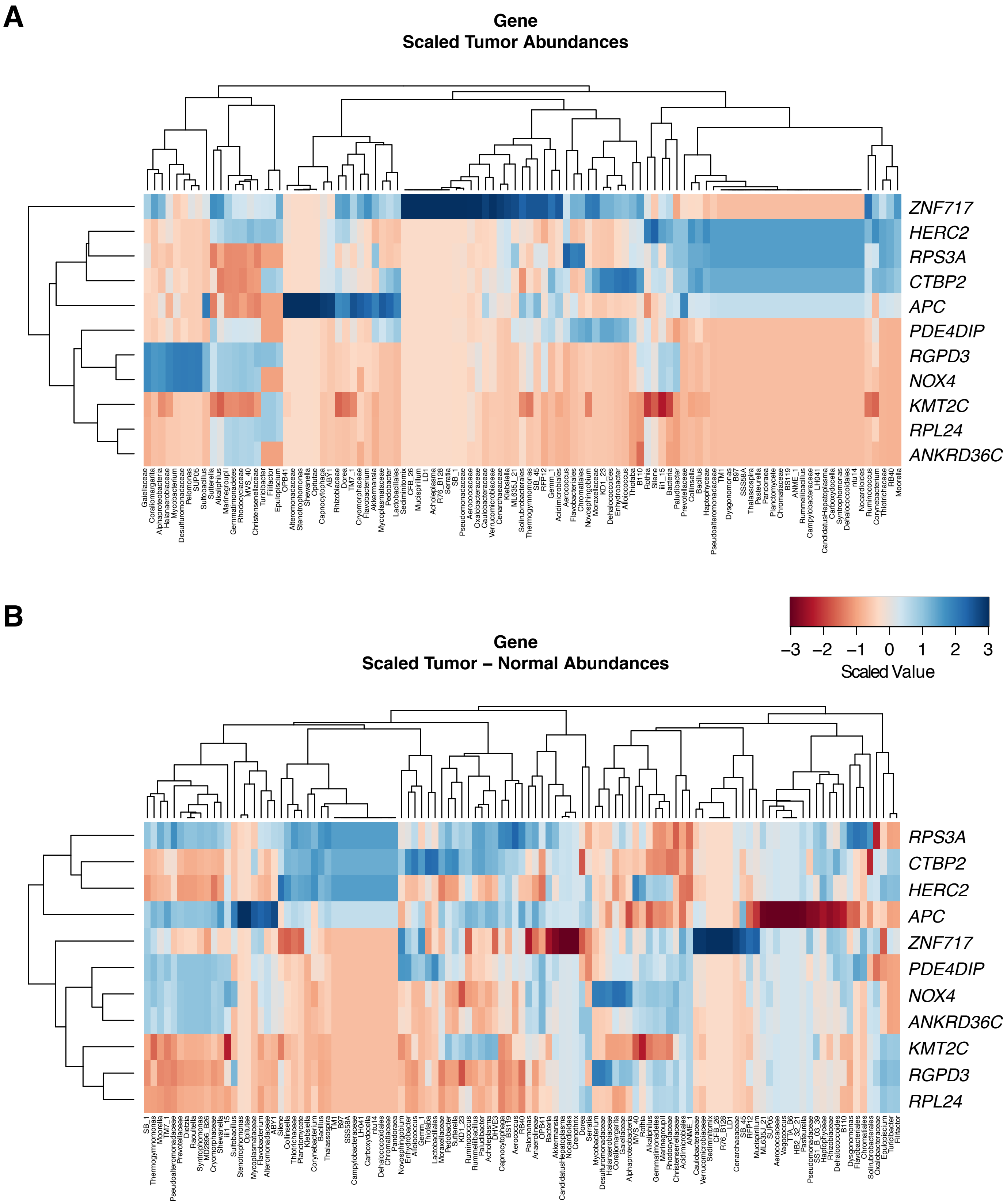

S5 Fig. Heatmaps demonstrating the patterns of microbial abundances for patient samples with prevalent LoF mutations. (A) Scaled taxon abundances (columns) in the tumor samples that harbor LoF mutations in the genes indicated (rows). (B) Scaled differences (tumor abundance - matched normal abun- dance) patients that harbor tumor-specific LoF mutations in the genes indicated (rows).
