# Supplementary material for: Colorectal cancer mutational profiles correlate with defined microbial communities in the tumor microenvironment
Source: PLoS Genet. 2018 Jun 20;14(6):e1007376. doi: 10.1371/journal.pgen.1007376 (PMC6028121; doi:10.1371/journal.pgen.1007376)
Supplement: S7 Fig — (A) Scaled taxon abundances (columns) in the tumor samples that harbor LoF mutations in the PID pathways indicated (rows). (B) Scaled differences (tumor abundance—matched normal abundance) patients that harbor tumor-specific LoF mutations in the PID pathways indicated (rows). (PDF) [file pgen.1007376.s023.pdf]

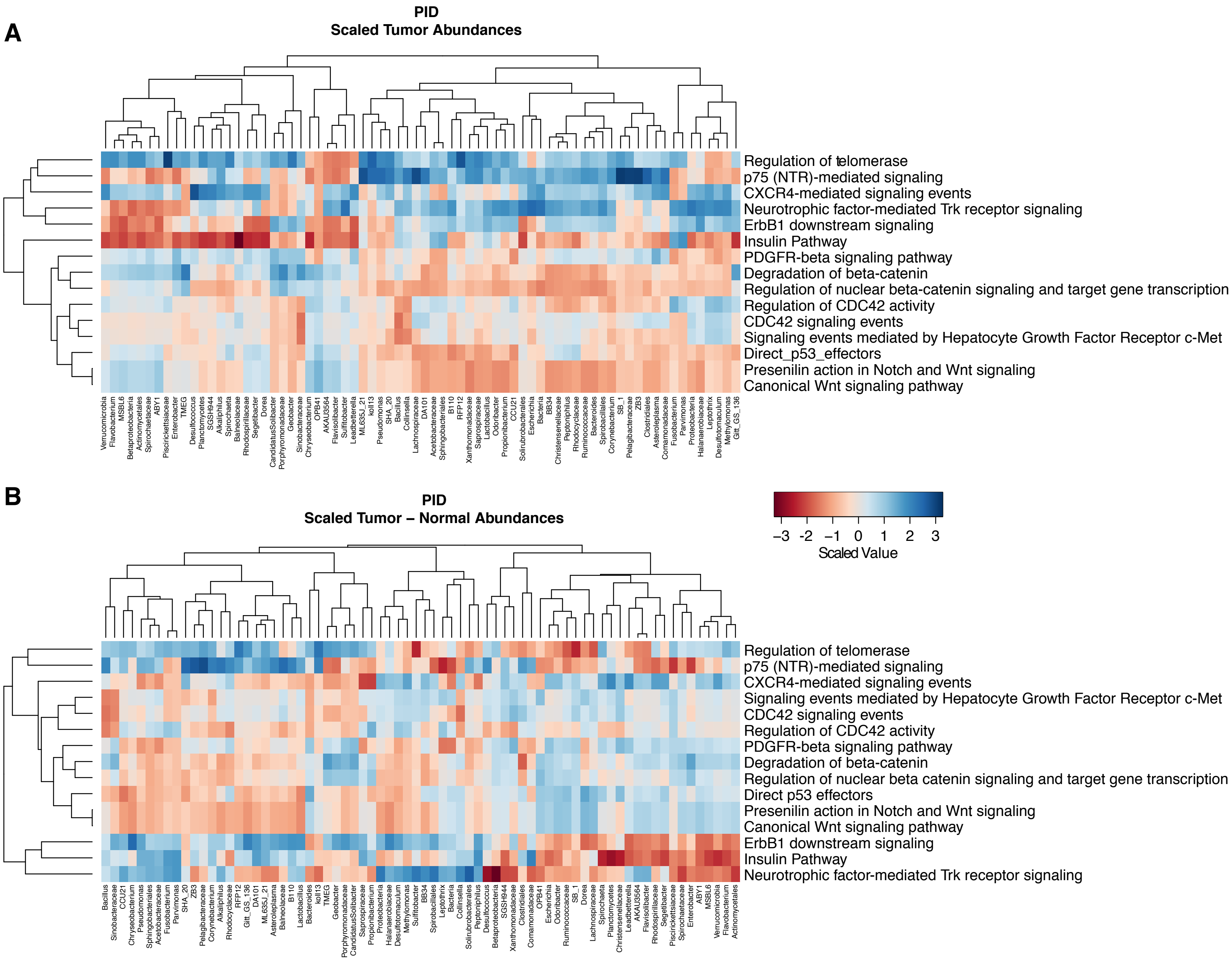

S7 Fig. Heatmaps demonstrating the patterns of microbial abundances for patient samples with prevalent LoF mutations in PID pathways. (A) Scaled taxon abundances (columns) in the tumor samples that harbor LoF mutations in the PID pathways indicated (rows). (B) Scaled differenc-es (tumor abundance - matched normal abundance) patients that harbor tumor-specfic LoF mutations in the PID pathways indicated (rows).
